# Application of a Mathematical Model to Describe the Effects of Chlorpyrifos on *Caenorhabditis elegans* Development

**DOI:** 10.1371/journal.pone.0007024

**Published:** 2009-09-15

**Authors:** Windy A. Boyd, Marjolein V. Smith, Grace E. Kissling, Julie R. Rice, Daniel W. Snyder, Christopher J. Portier, Jonathan H. Freedman

**Affiliations:** 1 Biomoleclular Screening Branch, National Toxicology Program, Research Triangle Park, North Carolina, United States of America; 2 SRA International, Durham, North Carolina, United States of America; 3 Biostatistics Branch, National Institute of Environmental Health Sciences, National Institutes of Health (NIH), Research Triangle Park, North Carolina, United States of America; 4 Laboratory of Molecular Toxicology, National Institute of Environmental Health Sciences, National Institutes of Health (NIH), Research Triangle Park, North Carolina, United States of America; Massachusetts General Hospital/Harvard Medical School, United States of America

## Abstract

**Background:**

The nematode *Caenorhabditis elegans* is being assessed as an alternative model organism as part of an interagency effort to develop better means to test potentially toxic substances. As part of this effort, assays that use the COPAS Biosort flow sorting technology to record optical measurements (time of flight (TOF) and extinction (EXT)) of individual nematodes under various chemical exposure conditions are being developed. A mathematical model has been created that uses Biosort data to quantitatively and qualitatively describe *C. elegans* growth, and link changes in growth rates to biological events. Chlorpyrifos, an organophosphate pesticide known to cause developmental delays and malformations in mammals, was used as a model toxicant to test the applicability of the growth model for *in vivo* toxicological testing.

**Methodology/Principal Findings:**

L1 larval nematodes were exposed to a range of sub-lethal chlorpyrifos concentrations (0–75 µM) and measured every 12 h. In the absence of toxicant, *C. elegans* matured from L1s to gravid adults by 60 h. A mathematical model was used to estimate nematode size distributions at various times. Mathematical modeling of the distributions allowed the number of measured nematodes and log(EXT) and log(TOF) growth rates to be estimated. The model revealed three distinct growth phases. The points at which estimated growth rates changed (change points) were constant across the ten chlorpyrifos concentrations. Concentration response curves with respect to several model-estimated quantities (numbers of measured nematodes, mean log(TOF) and log(EXT), growth rates, and time to reach change points) showed a significant decrease in *C. elegans* growth with increasing chlorpyrifos concentration.

**Conclusions:**

Effects of chlorpyrifos on *C. elegans* growth and development were mathematically modeled. Statistical tests confirmed a significant concentration effect on several model endpoints. This confirmed that chlorpyrifos affects *C. elegans* development in a concentration dependent manner. The most noticeable effect on growth occurred during early larval stages: L2 and L3. This study supports the utility of the *C. elegans* growth assay and mathematical modeling in determining the effects of potentially toxic substances in an alternative model organism using high-throughput technologies.

## Introduction

The U. S. Environmental Protection Agency estimates that there are at least 10,000 chemicals that require testing to evaluate their potential threat to human and environmental health [1]. Due to the need to screen such a large number of chemicals, three government agencies; the Environmental Protection Agency, National Toxicology Program and the NIH Chemical Genomics Center; signed an agreement to transform predictive toxicity testing from mainly *in vivo* mammalian studies to tests using alternative species and *in vitro* high-throughput screens [2]. The goals are to (a) develop reliable assays using alternative organisms or cell-based assays, (b) collect high-quality data using those assays, and then (c) assess whether those data can predict human toxicity.

One alternative animal model that has proven useful in toxicological research is the nematode *Caenorhabditis elegans* [3]. A strength of *C. elegans* as a model organism is the high degree of evolutionary conservation in its biological processes [4]. In addition, many of the stress response pathways, including those induced by exposure to environmental chemicals, are well-conserved [5].

*C. elegans* are self-fertilizing hermaphrodites that produce hundreds of genetically-identical offspring over several days of adulthood. *C. elegans* hatch into their first larval stage (L1) and continue to develop to adults through three additional distinct larval stages, L2-L4 [6]. Between each larval stage, nematodes grow in bursts by molting old cuticles [7]. *C. elegans* cultures can be synchronized by hatching embryos in the absence of food, causing L1 development to arrest [8]. The L1 larvae resume normal growth when food is introduced and mature as a synchronous population to gravid adults in approximately 60 h at 20°C [9].

Observations of up to 6,000 *C. elegans*/per minute can be made using the COPAS Biosort flow sorting system, which is designed to sort, dispense and measure various parameters of individual nematodes [10]. The Biosort measures and records up to four attributes for each individual: time of flight (TOF), which relates to nematode length; extinction (EXT), which corresponds to the optical density; and two fluorescence measurements. TOF and EXT measurements are related to the age and size of the nematode; both increase as *C. elegans* develop.

Assays and analytical methods have been developed that utilize the Biosort output to answer questions about *C. elegans* biology and the effects of toxicants [11]–[13]. A growth assay in which synchronized L1s are loaded into 96 well plates, incubated in the presence of toxicants, and their size distribution measured at later times has been developed. To accurately measure *C. elegans* growth in the presence and absence of toxicants, a mathematical model that describes changes in *C. elegans* size distributions as increases in EXT and TOF during nematode maturation was created [14]. A mathematical model was necessary to estimate *C. elegans* growth rates and size distributions due to the nature of the data generated by the Biosort, which were not directly amenable to rigorous statistical analysis. The Biosort provides EXT and TOF measurements of each nematode at loading (t = 0 h) and of the same set of nematodes at the end of the growth period. Initial measurements on an individual nematode, however, can not be matched to its final measurements at the end of the study. This type of data contrasts with growth measurements using cell culture, in which changes in the population are represented by single values (e.g., optical density, total cell number), or larger animal data in which changes in the growth (e.g., weight, height) can be assigned to an individual subject. Therefore, the *C. elegans* growth model mathematically describes the distributions of measurements on a set of nematodes, allowing means and average growth rates to be calculated.

One feature of the Biosort data that also needed to be addressed by the model was the presence of extraneous material such as shed cuticles or clumps of bacteria, which accumulate as *C. elegans* develop over time. Measurements on this extraneous material can not be automatically distinguished from those on the nematodes, and thus could affect statistical analyses. By mathematically modeling the distribution of measurements on the extraneous material, the model accounts for the extraneous matter and allows for a more accurate analysis of nematode growth.

In the present report, the growth model has been applied to test its effectiveness in detecting statistically significant differences in *C. elegans* development in the presence of a toxicant. The output from the analysis includes the effects of toxicant concentration and exposure time on three phases of *C. elegans* growth. To test and refine the growth assay, *C. elegans* were exposed to the environmentally-relevant, developmental neurotoxicant: chlorpyrifos. Chlorpyrifos is one of the most commonly applied organophosphate pesticides [15]. Organophosphate pesticides constitute almost half of all of the insecticides used worldwide [16]. In addition to its activity as a cholinesterase inhibitor, data suggest that chlorpyrifos may cause decreased DNA synthesis and developmental alterations [17], [18]. Growth retardation has also been observed in children exposed to chlorpyrifos *in utero* [19]. Due to adverse human and environmental health effects, chlorpyrifos has been banned for use in homes, schools, and hospitals [20].

EXT and TOF measurements on nematodes exposed to various chlorpyrifos concentrations were sampled over a 72 h period. Estimated numbers of nematodes and average growth rates based on EXT and TOF measurements were then calculated using the model. These estimates showed significant decreases in both numbers of nematodes and their growth rates as a function of chlorpyrifos concentration. In addition, L2 and L3 larvae were the most sensitive to chlorpyrifos exposure.

## Results

### *C. elegans* growth and model

Data from the two chlorpyrifos exposure studies were combined and analyzed using the *C. elegans* Markov growth model [14]. Each study consisted of thirty-six cohorts each containing 300 L1 nematodes. Six cohorts were exposed to one of five chlorpyrifos concentrations or an untreated control. Following 12, 24, 36, 48, 60, and 72 h incubations at 20°C, one cohort at each concentration was measured in the Biosort to determine its distribution of EXT and TOF.

**Figure 1:**
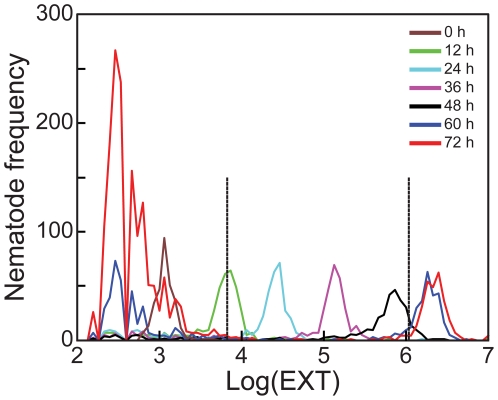
*C. elegans* growth from L1 to adult. Nematode frequency distributions of log(EXT) values for control nematode cohorts sampled at loading 0 h (*brown*) and following 12 (*green*), 24 (*light blue*), 36 (*purple*), 48 (*black*), 60 (*dark blue*), and 72 (*red*) h incubations. Large modes to the left of the loaded nematodes (log(EXT) ≈2–3) indicate the second generation of embryos corresponding to 60 and 72 h cohorts. Vertical lines at log(EXT)  = 3.83 and 6.04 divide the growth response into three sections: initial growth from starved L1s, larval growth from L2 to L4, and adult growth, respectively.

Distributions of log(EXT) at each of the six time points for the control cohorts are presented in Figure 1. These distributions shifted to the right with increasing incubation time; L1s loaded at t = 0 h had a peak frequency at log(EXT) value near 3.1, while nematodes observed following 60 h and 72 h incubations had peak frequency at log(EXT) values near 6.3. Optical density, as measured by EXT, tends to increase as nematodes mature [10]. Microscopic observations showed that L1 nematodes matured to L2s by 12 h, L3s by 24 h, L4s by 48 h, and adults by 60 h. After 60 h, adults showed a small increase in optical density but mainly produced offspring. The large numbers of measurements with log(EXT) values between 2 to 3.4 observed at 60 h and 72 h included unhatched embryos laid by the adults and the next generation of L1 larvae.

Under normal conditions *C. elegans* growth was thought to be approximately exponential [21]; therefore, growth rates with respect to either log(EXT) or log(TOF) should be fairly constant. We previously reported, however, that the best fitting model for either log(EXT) or log(TOF) contained three separate growth rates, separated by two change points [14]. The three sections in control nematodes are indicated in Figure 1 by vertical lines: log(EXT)  = 3.83 and 6.04. Microscopic observations indicated that these sections contained embryos and L1s (2–3.83); L2, L3 and L4 larvae (3.83–6.04); and adults (6.04–7.00).

### Effect of chlorpyrifos on the nematode frequency distribution

**Figure 2:**
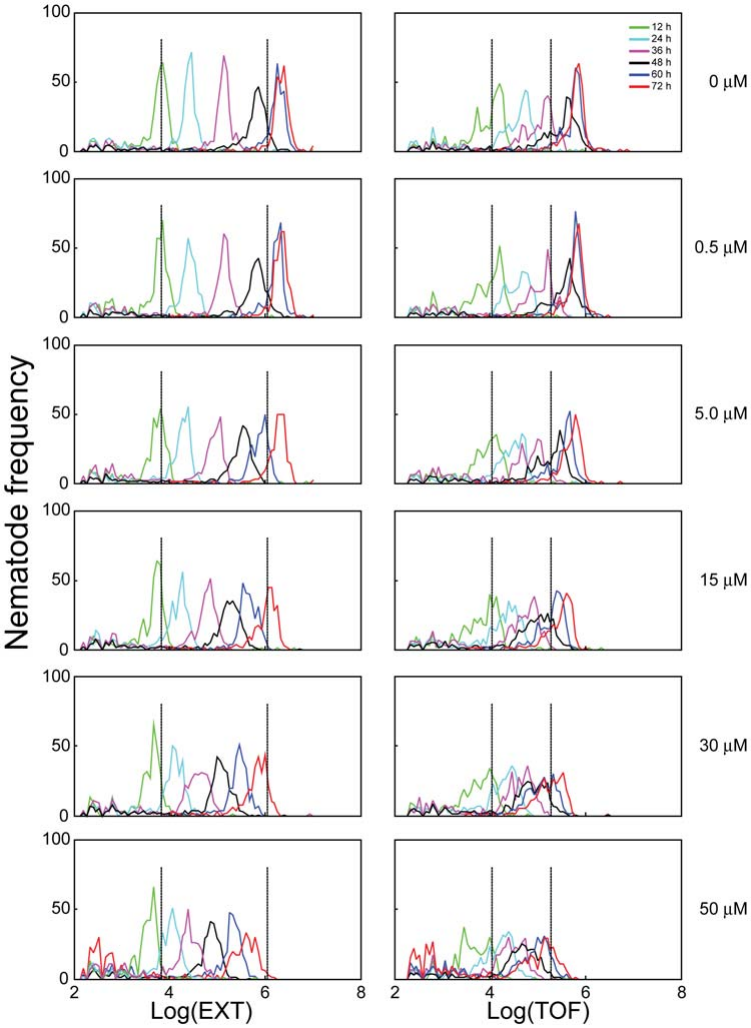
Effects of chlorpyrifos on *C. elegans* growth. Frequency distributions of log(EXT) (*left column*) and log(TOF) (*right column*) on nematodes exposed to 0, 0.5, 5, 15, 30, and 50 µM chlorpyrifos following 12 (*green*), 24 (*light blue*), 36 (*purple*), 48 (*black*), 60 (*dark blue*), and 72 (*red*) h incubations. Vertical lines indicate change points at log(EXT)  = 3.83 and 6.04 and log(TOF)  = 4.04 and 5.28.

Chlorpyrifos was used to study the applicability of the mathematical model in determining the effects of chemicals on *C. elegans* growth. Two experiments using different ranges of sub-lethal chlorpyrifos concentrations, 0–50 and 0–75 µM, were performed and the data combined to determine the effects on growth. The lowest concentration (0.5 µM) did not affect growth compared to untreated controls and the highest concentration (75 µM) completely inhibited growth, as determined by microscopic observation. Frequency distributions for log(EXT) and log(TOF) at each time point for the first experiment are presented in Figure 2. The frequency distributions for the second experiment are in Supporting Information File S1.

At the lowest concentration of chlorpyrifos tested (0.5 µM), the frequency distribution at each time point was nearly identical to that observed in control nematodes, indicating that chlorpyrifos did not affect *C. elegans* development. Furthermore, the close similarity at 60 and 72 h suggested that control nematodes and those exposed to 0.5 µM chlorpyrifos had fully developed to reproductive adults by 60 h (Fig. 2 and Supporting Information File S1). Above 0.5 µM, concentration-dependent decreases in growth were observed. At the highest concentration tested, 75 µM, the distributions did not shift to the right with time (Supporting Information File S1), suggesting that 75 µM chlorpyrifos completely inhibited nematode growth.

Nematodes exposed to chlorpyrifos concentrations greater than 45 µM did not develop beyond the second change point within 72 h (Fig. 2, Supporting Information File S1). Microscopic observations made at 72 h verified that, at concentrations below 30 µM, nematodes developed to the adult stage, but were smaller in size. *C. elegans* exposed to concentrations greater than 30 µM did not reach adulthood and at 75 µM did not develop beyond the L2 stage (Supporting Information File S1).

**Figure 3:**
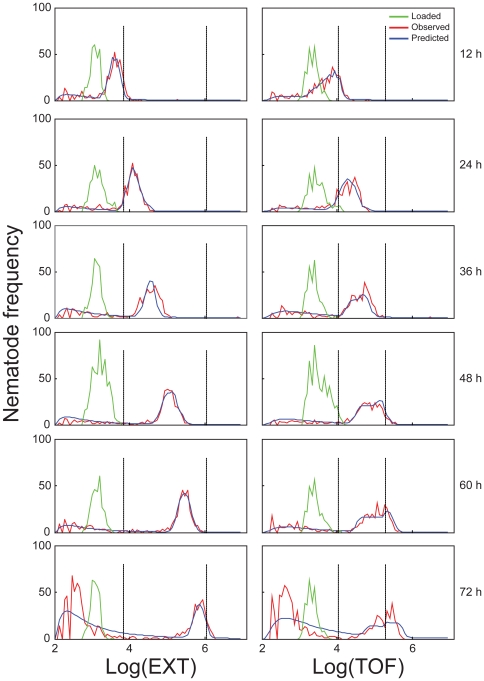
Observations and predictions across aspiration times using 30 µM chlorpyrifos. Observed (*red*) and model-estimated (*blue*) frequency distributions of log(EXT) (*left column*) and log(TOF) (*right column*). L1 nematodes loaded at the start of the experiment are also presented (*green*). Vertical lines indicate change points where growth rates changed at log(EXT)  = 3.83 and 6.04 and log(TOF)  = 4.04 and 5.28. Change points were constant over time and concentration, but differed between log(EXT) and log(TOF). Data for other chlorpyrifos concentrations are shown in Supporting Information Files S2 (log(EXT)) and S3 (log(TOF)).

The observed and model-estimated frequency distributions of log(EXT) and log(TOF) values for cohorts exposed to 30 µM are presented in Figure 3. Plots for other concentrations are presented in Supporting Information File S2 for log(EXT) and Supporting Information File S3 for log(TOF) measurements. Extraneous matter; such as detritus, clumps of bacteria, and embryos; can not be distinguished from nematodes directly from log(EXT) and log(TOF) measurements. For this reason, measurements were modeled using a mixture of a Markov model for the nematodes and a lognormal distribution for the extraneous matter (see Methods). This mixture model accurately described the distributions of log(TOF) and log(EXT) at each time point (i.e., compare red lines versus blue lines in Figure 3). At all exposure levels and time points, model-predicted frequency distributions agreed well with the observed frequency distributions. (It should be noted that the word prediction is used in this paper to refer to estimates made from the model after optimization, much as any calculated output from any model may be referred as a prediction. It does not refer to predictions made for use with other data or in different circumstances. The value of the model for the analysis in this paper lies in the estimation of growth rates of nematodes over time under specific conditions, including exposures to possible toxicants.)

### Effect of chlorpyrifos on *C. elegans* growth

**Figure 4:**
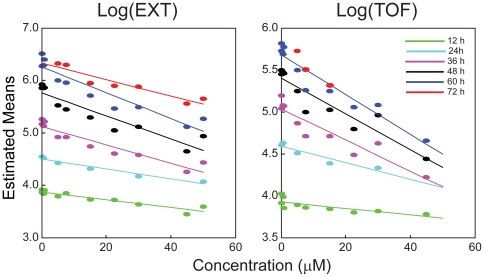
Effects of chlorpyrifos on mean *C. elegans* growth. Estimated means of log(EXT) (*left panel*) and log(TOF) (*right panel*) as functions of chlorpyrifos concentration; 0, 0.5, 0.75, 5.0, 7.5, 15, 22.5, 30, 45 and 50 µM; at six sampling times; 12 (*green*), 24 (*light blue*), 36 (*purple*), 48 (*black*), 60 (*dark blue*), and 72 (*red*) h. Statistical significance of concentration effect was tested by fitting straight lines through estimated means and testing slopes. A regression line for the 72 h log(TOF) data is not presented because there was no significant growth between 60 h and 72 h.

Although the chlorpyrifos concentrations were chosen to be sub-lethal, the model-estimated numbers of nematodes significantly decreased with increasing concentration (Page test; p<0.001). Estimated numbers of nematodes, as well as all other parameter estimates, are presented in Supporting Information File S4. The estimated mean log(EXT) and log(TOF) as functions of chlorpyrifos concentration and observation times are presented in Figure 4. These means diminished significantly as a function of chlorpyrifos concentration at each time point (by linear regression, p<0.01 for log(EXT); p<0.05 for log(TOF)) (Table 1). The strength of the concentration effects, as indicated by the magnitude of the slopes, also increased with exposure time. Taken together, these observations indicate that chlorpyrifos inhibits *C. elegans* growth in a concentration- and exposure time-dependent fashion.

**Table 1:** Slopes of mean log(EXT) and log(TOF) as a function of concentration by sampling time.

Sample (hour)	Log(EXT)	Log(TOF)
12	−0.0074	−0.0039
24	−0.0095	−0.0098
36	−0.0175	−0.0185
48	−0.0221	−0.0214
60	−0.0245	−0.0236
72	−0.0157	−0.0379*

* not significantly different from 0 at p<0.05; all other slopes were significant at p<0.01, except the 12 and 24 h log(TOF) measurements that are significant at a 0.05 level. The estimated means are shown in Figure 4.

For nematodes exposed to 0 and 0.5 µM chlorpyrifos, growth was completed by 60 h, so neither the TOF nor EXT measurement changed between 60 and 72 h (Fig. 2). Likewise, nematodes exposed to 75 µM chlorpyrifos grew very little over time, based on the extensive overlap in the distributions of log(TOF) among the time points (Supporting Information File S1). Previously, it was demonstrated that TOF measurements are generally more variable than EXT, and thus have a greater tendency to overlap [14]. In these cases where there is little to no change in TOF or EXT, growth rates can not be estimated by the mathematical model.

The growth model predicts three separate constant growth rates with respect to log(EXT) and log(TOF) measurements. The two points at which the growth rate changes, ‘change points’, are marked by vertical lines in Figures 1, 2, and 3; and divide the log(EXT) and log(TOF) ranges into 3 sections. Varying the change points for nematodes exposed to different chlorpyrifos concentrations did not improve the model fit; therefore change points were held constant for all concentrations at 3.83 and 6.04 for log(EXT) and at 4.04 and 5.28 for log(TOF).

**Figure 5:**
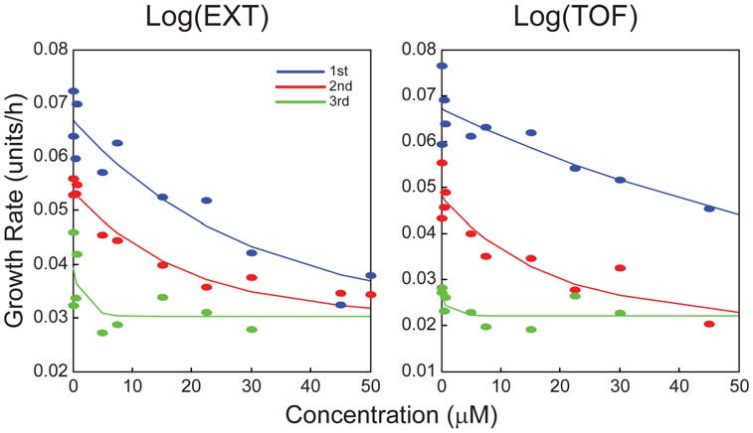
Growth rates of *C. elegans* after chlorpyrifos exposure. Estimated growth rates of log(EXT) per h (*left panel*) and log(TOF) per h (*right panel*) as functions of chlorpyrifos concentration. Growth rates are shown for three sections: initial growth rates before the first change point (*blue*), growth rates between change points (*red*), and growth rates after the second change point (*green*). Solid lines correspond to negative exponential functions with a common lower asymptote fit to the estimated growth rates. Nematodes exposed to chlorpyrifos concentrations greater than 30 µM did not grow to the third section.

To analyze the effects of chlorpyrifos on the estimated growth rates, a negative exponential function, 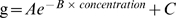, was fit to the growth rates in each of the three sections as functions of concentration (Fig. 5; equations for each section are presented in Supporting Information File S5). The strength of the chlorpyrifos effect on the growth rate is indicated by *B*. The final models for the three sections were chosen using the Akaike information criterion [22]. For every section, *B* was significantly greater than zero, indicating a significant inhibitory effect of chlorpyrifos on growth. In addition, the effect increased with increasing chlorpyrifos concentration for each of the three sections, further indicating concentration dependent growth inhibition (Fig. 5; Table 2). The functions eventually reached the same minimum growth rate for all three sections (Fig. 5), which may represent the minimum growth rate that the model could detect.

**Table 2:** Growth rates at each chlorpyrifos concentration.

	Log(EXT)	Log(TOF)
Concentration (µM)	Section 1 (2.00, 3.83)	Section 2 (3.83, 6.04)	Section 3 (6.04, 7.00)	Section 1 (2.00, 4.04)	Section 2 (4.04, 5.28)	Section 3 (5.28, 7.00)
0	0.0639	0.0529	0.0323	0.0765	0.0433	0.0283
0	0.0723	0.0559	0.0460	0.0593	0.0553	0.0272
0.5	0.0596	0.0531	0.0337	0.0691	0.0457	0.0232
0.75	0.0698	0.0548	0.0419	0.0639	0.0489	0.0261
5.0	0.0571	0.0455	0.0272	0.0612	0.0400	0.0229
7.5	0.0626	0.0445	0.0287	0.0631	0.0351	0.0197
15	0.0525	0.0398	0.0339	0.0619	0.0347	0.0192
22.5	0.0518	0.0358	0.0311	0.0541	0.0278	0.0264
30	0.0422	0.0376	0.0279	0.0517	0.0325	0.0227
45	0.0325	0.0346		0.0454	0.0203	
50	0.0380	0.0343				

Empty boxes indicate insufficient number of observations to estimate growth rates because nematodes did not grow sufficiently for model predictions.

**Figure 6:**
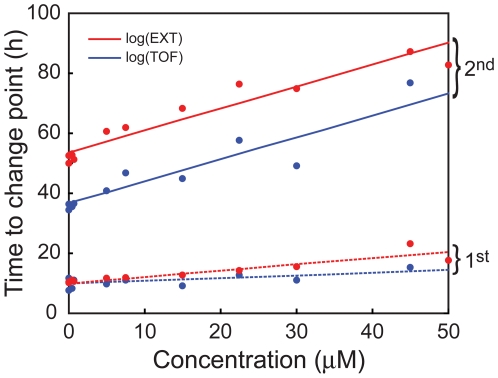
Effect of chlorpyrifos on the estimated times to change points. Regression lines correspond to the time required for the *C. elegans* cohort to develop to the first (*dotted line*) or the second (*solid line*) change point for log(EXT) (*red*) and log(TOF) (*blue*) values. For the first change point, log(EXT) and log(TOF) times at lower chlorpyrifos concentrations (0.5 and 0.75 µM) are indistinguishable from control nematodes while, at higher concentrations, log(EXT) was more affected than log(TOF). In contrast, the difference in time to reach the second change point between log(TOF) and log(EXT) is 17 h, regardless of chlorpyrifos concentration.

The expected length of time needed for a nematode to reach each change point was computed from the estimated growth rates (Fig. 6), and then straight lines were fit as functions of concentration. The final models for both change points were chosen using the Akaike information criterion [22]. The expected time for L1s to develop and reach the first change point was similar in the controls for both log(EXT) and log(TOF), approximately 10 h, and increased significantly with chlorpyrifos concentration. Expected length of time to reach the first change point, based on optical density (log(EXT)), was somewhat more affected by increasing chlorpyrifos concentration than that based on nematode length (log(TOF)), as indicated by the steeper slope (Fig. 6). These results are consistent with the visual observations that chlorpyrifos-exposed nematodes appeared to be starved and thinner than controls, but not shorter in length (Table 3). The expected length of time for L1s to develop to the second change point also showed a concentration-dependent increase for both log(EXT) and log(TOF). These observations further demonstrate the inhibitory effects of chlorpyrifos on growth. In contrast to the time to reach the first change point, for the second change point there was a consistent 17 h difference between log(TOF) and log(EXT), regardless of chlorpyrifos concentration.

**Table 3:** Visual observations made near change points.

**A. First change point (indicative of L1 → L2 molt)**										
**Concentration**	**Time**			**Expected times to change point**				**Model-predicted stages**		**Observed stages**
				TOF	EXT					
0 µM	12 h			11 h	10 h			mostly L2s		Well-fed L2s
45 µM	12 h			15 h	23 h			mostly L1s		Starved L2s
**B. Second change point for log(TOF) (indicative of L3 → L4 molt)**										
**Concentration**	**Time**			**Expected time to change point**			**Model-predicted stages**		**Observed stages**	
0 µM	36 h			34 h			mostly L4s		Active L3s	
0.75 µM	36 h			36 h			Mix of L3s/L4s		Active L3s	
7.5 µM	36 h			46 h			Mix of L2s/L3s		Slower L3s	
7.5 µM	48 h			47 h			Mix of L3s/L4s		Inactive L3s	
22.5 µM	48 h			57 h			Mix of L2s/L3s		Slower, smaller L3s	
22.5 µM	60 h			57 h			mostly L4s		Starved L4s	
45 µM	48 h			77 h			Mix of L2s/L3s		Inactive L2s	
45 µM	60 h			76 h			Mix of L2s/L3s		Small L3s	
**C. Second change point for log(EXT) (indicative of egg production)** *										
**Concentration**		**Time**	**Expected time to change point**			**Model-predicted stages**			**Observed stages**	
0 µM		48 h	50 h			mostly L4s			Active L4s	
0.75 µM		60 h	50 h			Gravid adults			Young adults, eggs	
7.5 µM		48 h	51 h			Mix of L4s/young adults			Active L4s	
7.5 µM		60 h	51 h			Gravid adults			Thinner adults than control; eggs	
22.5 µM		60 h	62 h			Mix of L4s/young adults			L4s	
22.5 µM		72 h	76 h			Mix of L4s/young adults			Lighter and smaller adults, very few eggs	

* Data for 45 µM chlorpyrifos is not presented because the expected time to reach the second change point was >80 h, which was beyond the end of the experiment.

### Effect of chlorpyrifos on change points

Although numbers of nematodes and their growth rates were affected by chlorpyrifos concentration, the positions of the estimated change points for both log(EXT) and log(TOF) were unaffected. That is, the rates for the three phases of *C. elegans* growth were affected by chlorpyrifos, but the log(EXT) and log(TOF) values during *C. elegans* development at which growth rates changed remained the same. Microscopic observations of non-exposed nematodes at the change points indicated that the first change point coincided with the L1 to L2 molt, while the second change point occurred near the L3 to L4 molt for log(TOF), and at the onset of egg production in young adults for log(EXT) values (Table 3).

Microscopic observations of selected chlorpyrifos-exposed nematodes were made at times corresponding to the change points: observations made at the times corresponding to first change point for both EXT and TOF (Table 3A); observations made near the expected time for the second log(TOF) change point (Table 3B); and observations made near the expected time for the second change point for the log(EXT) measurements (Table 3C). While chlorpyrifos increased the time to reach each change point, it did not substantially alter the composition of the cohort (Table 3B and 3C). Thus, the growth rates during these phases of *C. elegans* growth were affected by chlorpyrifos, but the biological events that occurred at the three change points during development were not affected by chlorpyrifos. Combining the information on the effects of chlorpyrifos on the time required to reach the change points (Fig. 6) with the visual observations of *C. elegans* at the change points (Table 3) led to the conclusion that the greatest developmental effect of chlorpyrifos was to increase the duration of the L2 and L3 larval stages and to delay the L3/L4 molt. For control nematodes, the length of time between the L1/L2 molt (first log(TOF) change point) and the L3/L4 molt (second log(TOF) change point) was approximately 27 h for control nematodes and was 52 h for nematodes exposed to 50 µM chlorpyrifos. In contrast, the length of time between the L3/L4 molt (second log(TOF) change point) and the onset of egg production (second log(EXT) change point) remained constant at about 17 h, regardless of level of exposure. Thus, once nematodes reached the L3/L4 molt, further development was not significantly affected by chlorpyrifos.

## Discussion

Chemical effects on *C. elegans* can be quantified using a variety of endpoints such as feeding behavior, locomotion, and egg-laying [11], [13], [23]. In this report, the applicability of a quantitative medium-throughput growth assay, analyzed using a mathematical model to determine the effects of chemicals on *C. elegans* was presented. These results confirmed the validity of this model, which provided information on chemical-induced changes in numbers of nematodes and growth rates. In addition, the concentration dependence of these effects was clearly demonstrated and quantified.

Many *C. elegans* studies have reported the measurement of abnormal growth phenotypes, mainly by measuring the delay to a predetermined developmental stage such as molting. The timing of molts, however, and the regulation of developmental rates within each larval stage have been poorly characterized [24]. Several studies have measured nematode length or volume using microscope reticles or image capture software [25], [26]. Although these methods are quantitative, they are tedious and can only be used to characterize small numbers of nematodes, and thus have limited statistical power. The COPAS Biosort is able to rapidly record measurements of length and optical density on large numbers of nematodes. Until now however, the data provided by the Biosort have not been quantitatively analyzed in a way that provides insight into the biology associated with chemical treatments. In applying the Markov model for growth [14] to nematodes exposed to the developmental toxicant chlorpyrifos, concentration effects can be accurately described. Once properly described, statistical tests for significance in changes in numbers of nematodes, mean log(EXT) or log(TOF) values, or estimated growth rates can be determined.

The optimal form of the model describes growth as piecewise linear over time, based on both log(EXT) and log(TOF). The times at which the growth rates change, referred to as change points, are associated with L1 to L2 molts for both log(EXT) and log(TOF), L3 to L4 molts for log(TOF), and the onset of egg production in young adults for log(EXT) [14]. Visual examination of nematodes near the estimated times for growth rate change in the current study verified that these characterizations are reproducible between different studies. In addition, while exposure to chlorpyrifos prolongs the times to reach the change points, it does not significantly change the composition of the cohort of nematodes at the change points. Thus, this model provides a link between rapidly produced Biosort data and biological events such as specific molts or onset of egg production.

The effect of chlorpyrifos exposure on the time required to reach a change point was not proportional to the toxicant concentration at all stages of development. The length of time between the L1/L2 molt and the L3/L4 molt increased from approximately 27 h for untreated nematodes to 52 h for nematodes exposed to 50 µM chlorpyrifos. The length of time between the L3/L4 molt and onset of egg production, however, was concentration independent and remained constant at 17 h. The difference between the second log(TOF) change point (L3/L4 molt) and the second log(EXT) change point (onset of egg production in adulthood) were consistent with published observations, which showed that at 20°C the L4/adult molt occurred approximately 13 h after the L3/L4 molt and the first embryos were laid 21.5 h after the L3/L4 molt [21]. Thus, in addition to characterizing an overall inhibition of nematode growth by chlorpyrifos, L2 and L3 larvae were identified as more susceptible to chlorpyrifos toxicity than later larvae or adults.

Exposure to chlorpyrifos in mammalian systems is associated with the inhibition of neuronal development [27]. Interestingly, the development of much of the ventral nervous system in *C. elegans* occurs during the early larval stages [28]. An additional 80 neurons (∼30% of the *C. elegans* nervous system) are born during L1 and L2 stages [28]. The growth assay may have also identified a relation between the growth inhibitory effects associated with chlorpyrifos exposure and neural development. This suggests that this assay may identify subtle effects of toxicant exposure during specific periods in *C. elegans* development. Additional studies will be required to confirm a neurodevelopmental affect of chlorpyrifos exposure.

*C. elegans* growth and development are known to be substantially affected by exposure to aldicarb, another cholinesterase inhibitor [29]. Prolonged exposure or exposure to high doses of aldicarb may also decrease feeding, leading to an indirect effect on nematode growth [26]. The effect of chlorpyrifos on *C. elegans* feeding, as measured by the accumulation of fluorescent-labeled microspheres, has been previously reported. A 50% inhibition of feeding at 1.3 µM chlorpyrifos (95% C.I.  = 0–9.2) was observed [11]. In this study, nematodes were observed with a ‘starved’ appearance following exposure to chlorpyrifos concentrations greater than 7.5 µM (Table 3). This suggests that starvation may be a contributing factor to chlorpyrifos-induced growth inhibition.

### Conclusion

A hallmark of high-throughput screening is automation of data collection, which rapidly produces large quantities of data. Linking quantitative data to biological events allows for the interpretation of trends and differences. In this report, high-throughput data from the Biosort was combined with a mathematical model of *C. elegans* growth. Modeling of *C. elegans* development allowed for the estimation of several growth parameters including number of nematodes, mean population distribution, and growth rates. Statistical analyses of these parameters showed concentration-dependent decreases in both numbers of nematodes and growth rates as a result of chlorpyrifos exposure. Although chlorpyrifos affected the time to reach the change points, the biological events that occurred at those points were not affected. In addition, the model indicated that the greatest chlorpyrifos effects occurred during larval stages L2 and L3.

The precise mechanism by which chlorpyrifos affected *C. elegans* growth could not be determined using these assays. Regardless of the mechanism of the developmental delay, if the effects of chemicals on *C. elegans* growth are predictive of mammalian toxicity, then *C. elegans* assays may prove to be valuable additions to *in vivo* high-throughput toxicity screens of the large number of chemicals that need to be assayed before mammalian testing.

## Methods

### Nematode culture

The Bristol N2 *C. elegans* strain was obtained from the Caenorhabditis Genetic Center (Minneapolis, MN), and maintained at 20°C on K-agar plates seeded with *E. coli* OP50 as a food source [30], [31]. Synchronized L1 cultures were prepared as previously described [8], except that embryos were hatched overnight at 20°C in vented T25 flasks containing complete K-medium (51 mM sodium chloride, 32 mM potassium chloride, 3 mM calcium chloride, 3 mM magnesium sulfate, 13 µM cholesterol).

### Growth assay

L1 nematodes were transferred to the sample cup of the COPAS Biosort [32]–[35] (Union Biometrica Inc., Somerville, MA, USA) and diluted to approximately 1 nematode/µL. Twenty-five L1s were then added to each well of a 96-well plate, containing a total volume of 50 µL complete K-medium, streptomycin-killed (1 mg/kg) *E. coli*, diluted to an A_550_  = 0.5–0.55, and chlorpyrifos (Chem Service, Westchester, PA; CAS#2921-88-2).

Chlorpyrifos stocks were prepared in DMSO. The final concentration of DMSO used in these studies was 1% (v/v), a concentration that does not affect nematode growth (data not shown). Two experiments were performed using six different chlorpyrifos concentrations with cohorts measured every 12 h for 72 h. A cohort consisted of 300 nematodes per treatment condition (25 nematodes per well×12 wells). Two different concentration series were tested in the two experiments: 0, 0.5, 5.0, 15, 30 and 50 µM, and 0, 0.75, 7.5, 22.5, 45 and 75 µM. *C. elegans* cohorts were incubated without shaking for 12, 24, 36, 48, 60 and 72 h at 20°C and then TOF and EXT measurements of individual nematode were acquired with the COPAS Biosort ReFLx. All COPAS Biosort measurements were performed with EXT signal gain of 80, EXT integral gain of 100, EXT signal threshold of 80, and TOF minimum of 10.

### Study design and model description

Both log(EXT) and log(TOF) measurements were analyzed using the Markov model applied to *C. elegans* growth previously described [14]. Briefly, a frequency distribution of measurements is constructed using a grid of bins encompassing the observed range of log(EXT) or log(TOF) values. Growth of nematodes is modeled by assuming that over each 12 h interval, nematodes grow sufficiently that their measurements shift to the right 10, 11, or 12 bins. The probabilities of growing 10, 11, or 12 bins are used to define a transfer probability or growth matrix, G. Using the distribution of measurements taken at loading, f_0_, the distribution of the same cohort at aspiration 12×k h later, f_k_, is predicted by the formula:
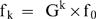
(1)


In addition to nematodes, extraneous material accumulated in the wells is also measured by the Biosort. These extraneous measurements, which may be discarded cuticles, clumped bacteria, or chemical precipitates, are not individually separable from nematode measurements. The extraneous measurements are represented in the model by a lognormal distribution. The total set of measurements acquired at sampling time k (nematodes and extraneous) is modeled as a weighted average of the lognormal distribution and the distribution, f_k_, predicted by the Markov model. The weights estimated for the lognormal and modeled distributions reflect the fractions of the total measurements ascribed to extraneous observations and nematodes, respectively.

The model allows 12 h growth across a constrained number of bins (i.e., 10, 11, or 12); growth rates are reflected by the size of the bins. Using least squares, the difference between observed and estimated frequency distributions of nematodes is minimized by adjusting bin sizes, where larger bins correspond to faster growth rates. In this study, changes in log(EXT) and log(TOF) measurements are best approximated by piecewise constant growth rates, with two change points dividing the range of measurements into three sections. In addition to estimating growth rates, the expected time for a nematode to reach a change point is calculated by averaging the time needed to shift the required number of bins to the right over the loading distribution.

### Statistical analysis

A distribution-free test for trend (Page test), blocking on observation time, was used to test whether concentration-related trends in numbers of nematodes were significant [36]. Application of the Page test requires a balanced design, so the estimated numbers of nematodes for the 72 h aspirations (available only for the higher concentrations) could not be used. The linear associations between exposure concentration and mean log(EXT), mean log(TOF) or time to change points were tested for significance using standard regression tests (F-tests; [37]). Negative exponential curves were fit to the estimated growth rates, and straight lines were fit to the estimated times to change points. Optimal models for growth rates and times to change points were chosen using the Akaike information criterion [22].

## Acknowledgments

Nematode strains used in this work were provided by the Caenorhabditis Genetics Center, which is funded by the NIH National Center for Research Resources. The authors thank Matthew McElwee for his thoughtful comments.

## Supporting Information

Supporting Information File S1 Frequency distributions of (a) log(EXT) and (b) log(TOF) on nematodes exposed to 0, 0.75, 7.5, 22.5, 45, and 75 µM chlorpyrifos at 12, 24, 36, 48, 60, and 72 h. Vertical lines divide the growth response into 3 sections: initial growth from starved L1s, larval growth from L2-L4, and adult growth.
(0.04 MB PDF)

Supporting Information File S2 Observed (red) and model-predicted (blue) distributions of log(EXT). The distribution of the loaded nematodes is shown in green. Extraneous noise was modeled as a lognormal
(0.08 MB PDF)

Supporting Information File S3 Observed (red) and model-predicted (blue) distributions of log(TOF). The distribution of the loaded nematodes is shown in green. Extraneous noise was modeled as a lognormal distribution (black). Black vertical lines indicate change points, where growth rates changed.
(0.08 MB PDF)

Supporting Information File S4 Tables
(0.07 MB PDF)

Supporting Information File S5 Negative exponential functions fit to growth rates.
(0.01 MB PDF)
