# Supplementary material for: Application of a Mathematical Model to Describe the Effects of Chlorpyrifos on Caenorhabditis elegans Development
Source: PLoS One. 2009 Sep 15;4(9):e7024. doi: 10.1371/journal.pone.0007024 (PMC2737145; doi:10.1371/journal.pone.0007024)
Supplement: Supporting Information File S1 — Frequency distributions of (a) log(EXT) and (b) log(TOF) on nematodes exposed to 0, 0.75, 7.5, 22.5, 45, and 75 µM chlorpyrifos at 12, 24, 36, 48, 60, and 72 h. Vertical lines divide the growth response into 3 sections: initial growth from starved L1s, larval growth from L2-L4, and adult growth. (0.04 MB PDF) [file pone.0007024.s001.pdf]

Supplementary File 1 - Observations of chlorpyrifos effects on *C. elegans* growth.

Frequency diagrams of (a) log(EXT) and (b) log(TOF) observations on nematodes exposed to 0, 0.75, 7.5, 22.5, 45, and 75 µM chlorpyrifos at 12, 24, 36, 48, 60, and 72 h. Vertical lines divide the growth response into 3 sections: initial growth from starved L1s, larval growth from L2-L4, and adult growth.

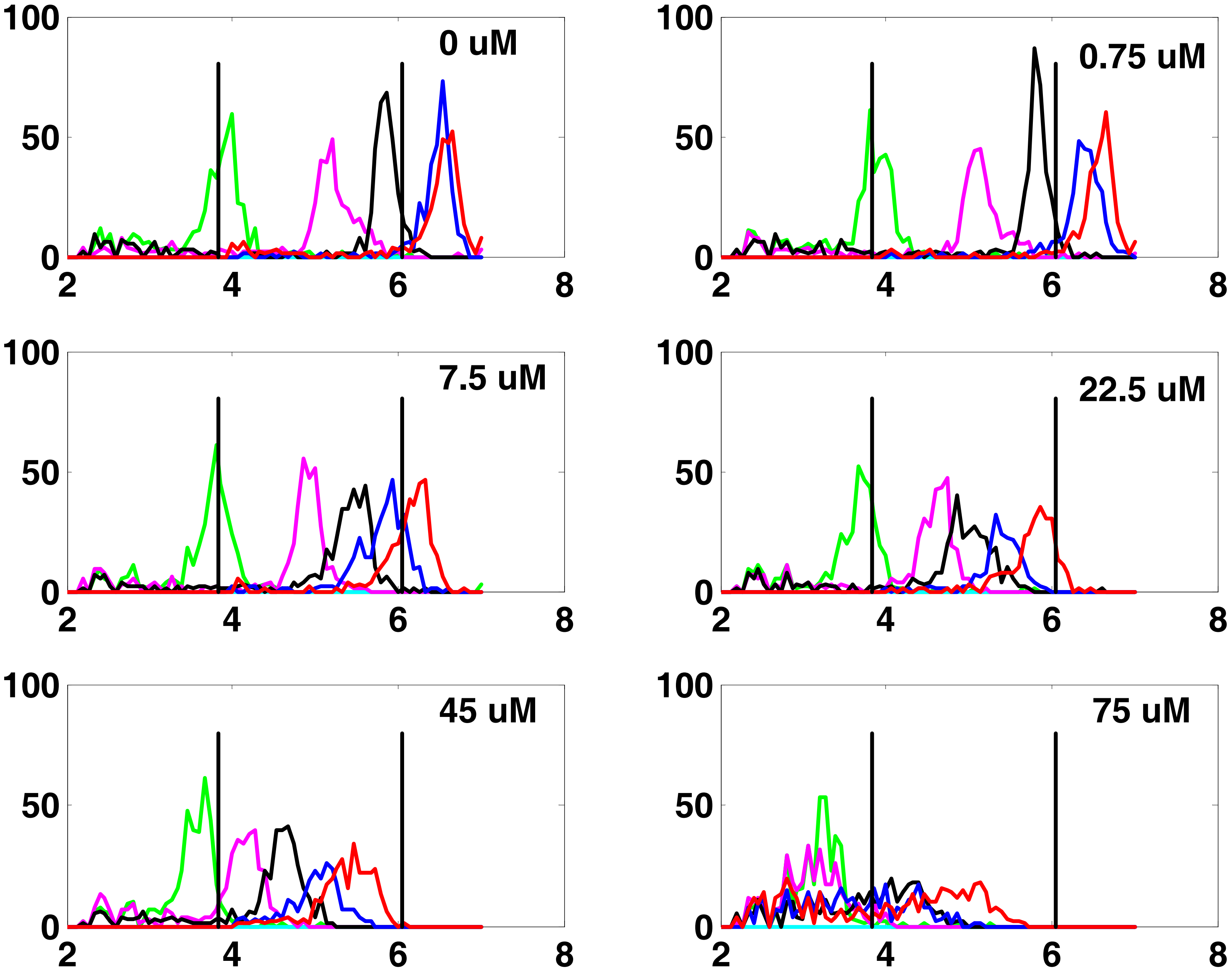

Suppl Fig 1a

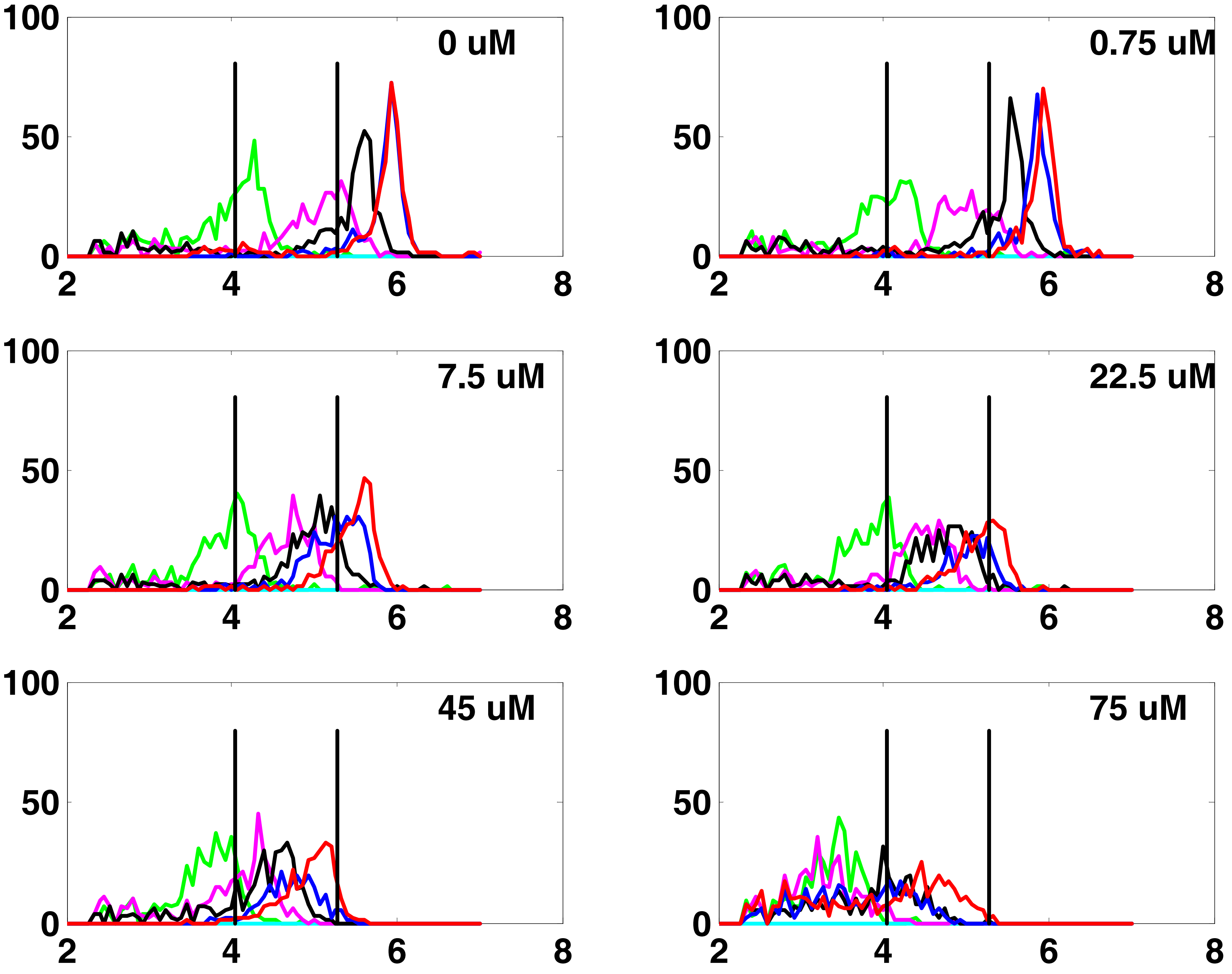

Suppl Fig 1b
