# Supplementary material for: Application of a Mathematical Model to Describe the Effects of Chlorpyrifos on Caenorhabditis elegans Development
Source: PLoS One. 2009 Sep 15;4(9):e7024. doi: 10.1371/journal.pone.0007024 (PMC2737145; doi:10.1371/journal.pone.0007024)
Supplement: Supporting Information File S2 — Observed (red) and model-predicted (blue) distributions of log(EXT). The distribution of the loaded nematodes is shown in green. Extraneous noise was modeled as a lognormal (0.08 MB PDF) [file pone.0007024.s002.pdf]

Supplementary File 2 - Observations and predictions of log(EXT) across all aspiration times and chlorpyrifos concentrations.

Observed frequencies (log(EXT)) of all aspirated measurements (red) and model estimated frequencies of maturing nematodes (blue). The distribution of the loaded nematodes is shown in green. Extraneous noise was modeled as a lognormal distribution (black). Black vertical lines indicate change points, where growth rates changed.

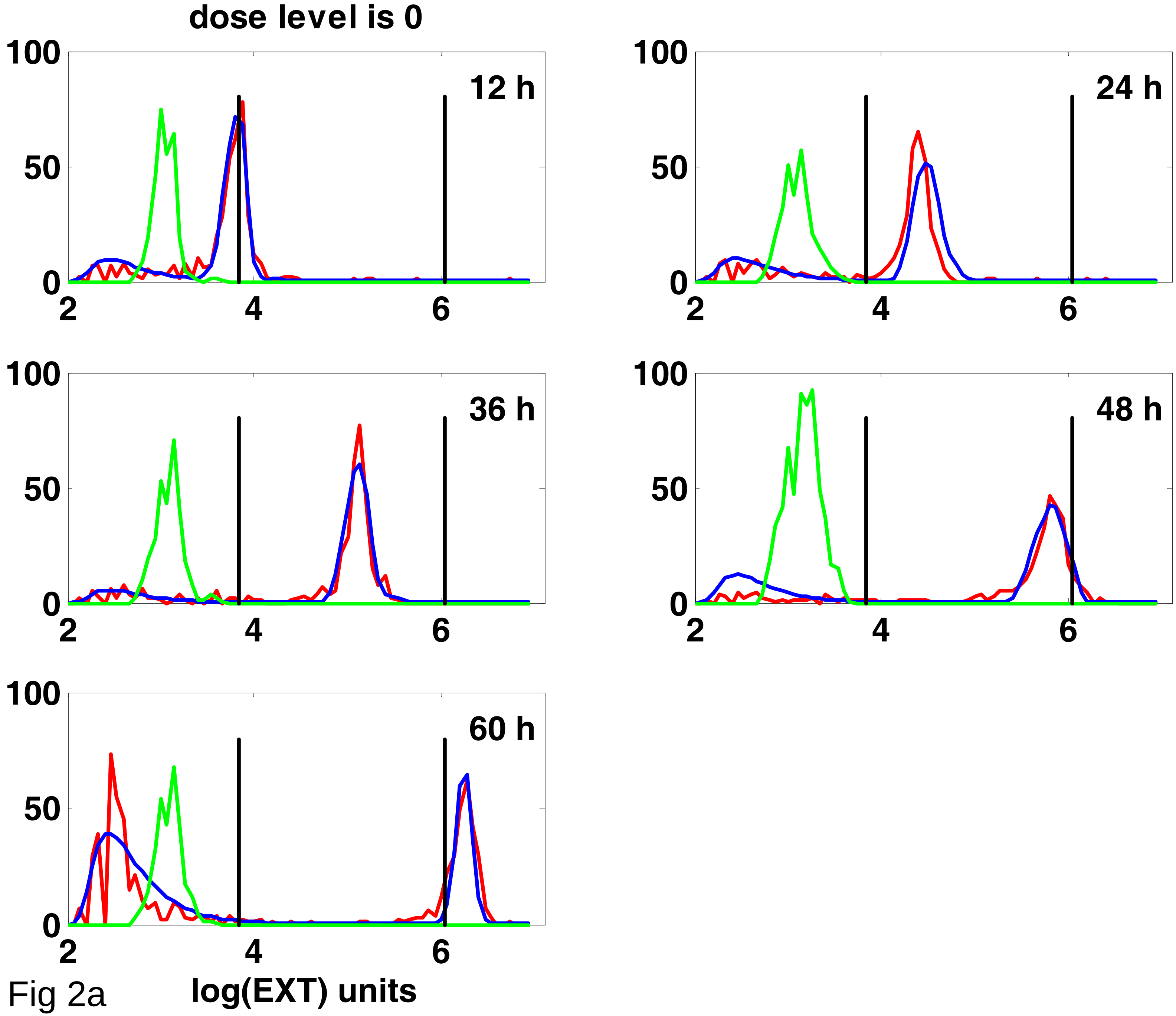

Suppl Fig 2a

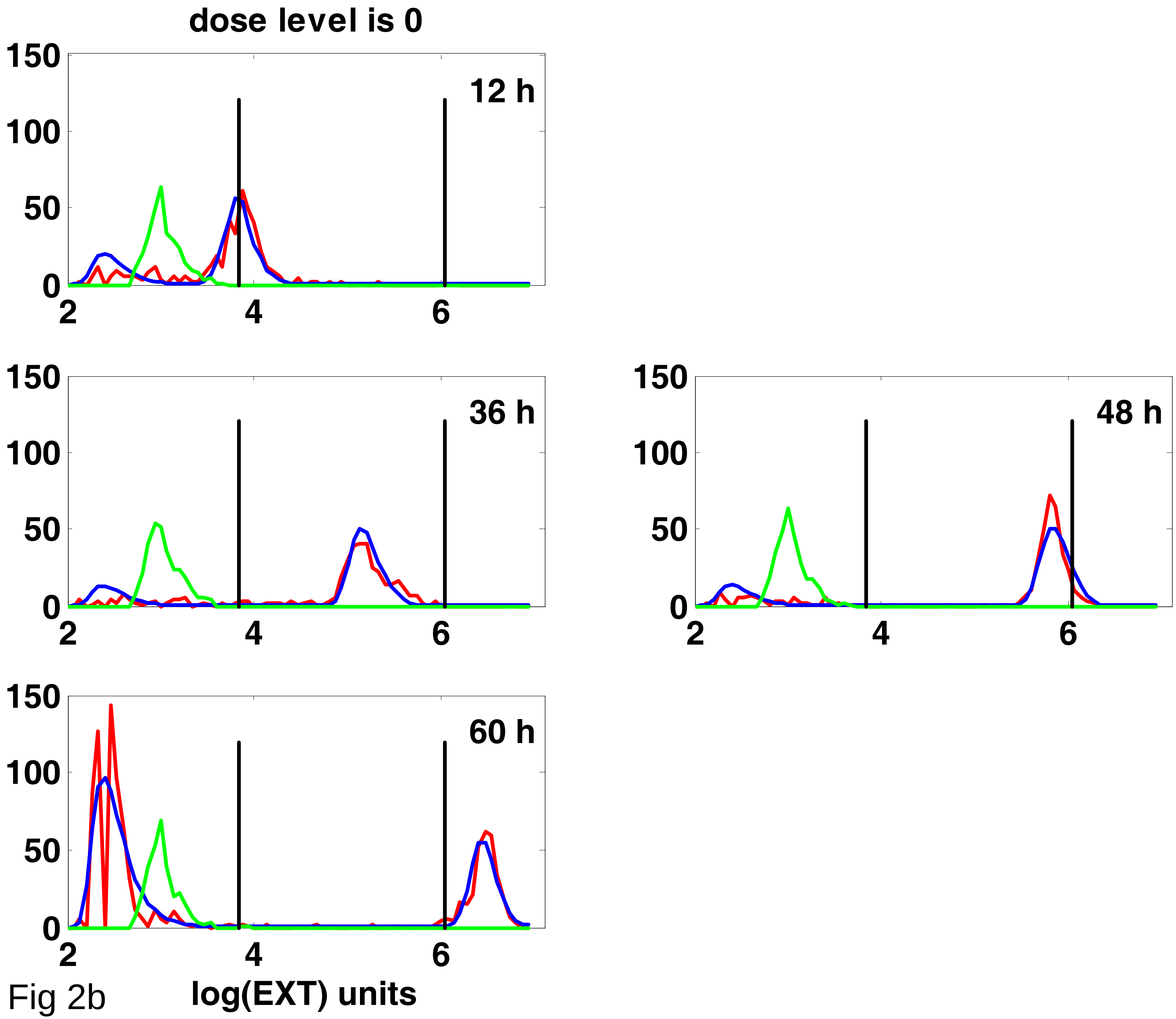

Suppl Fig 2b

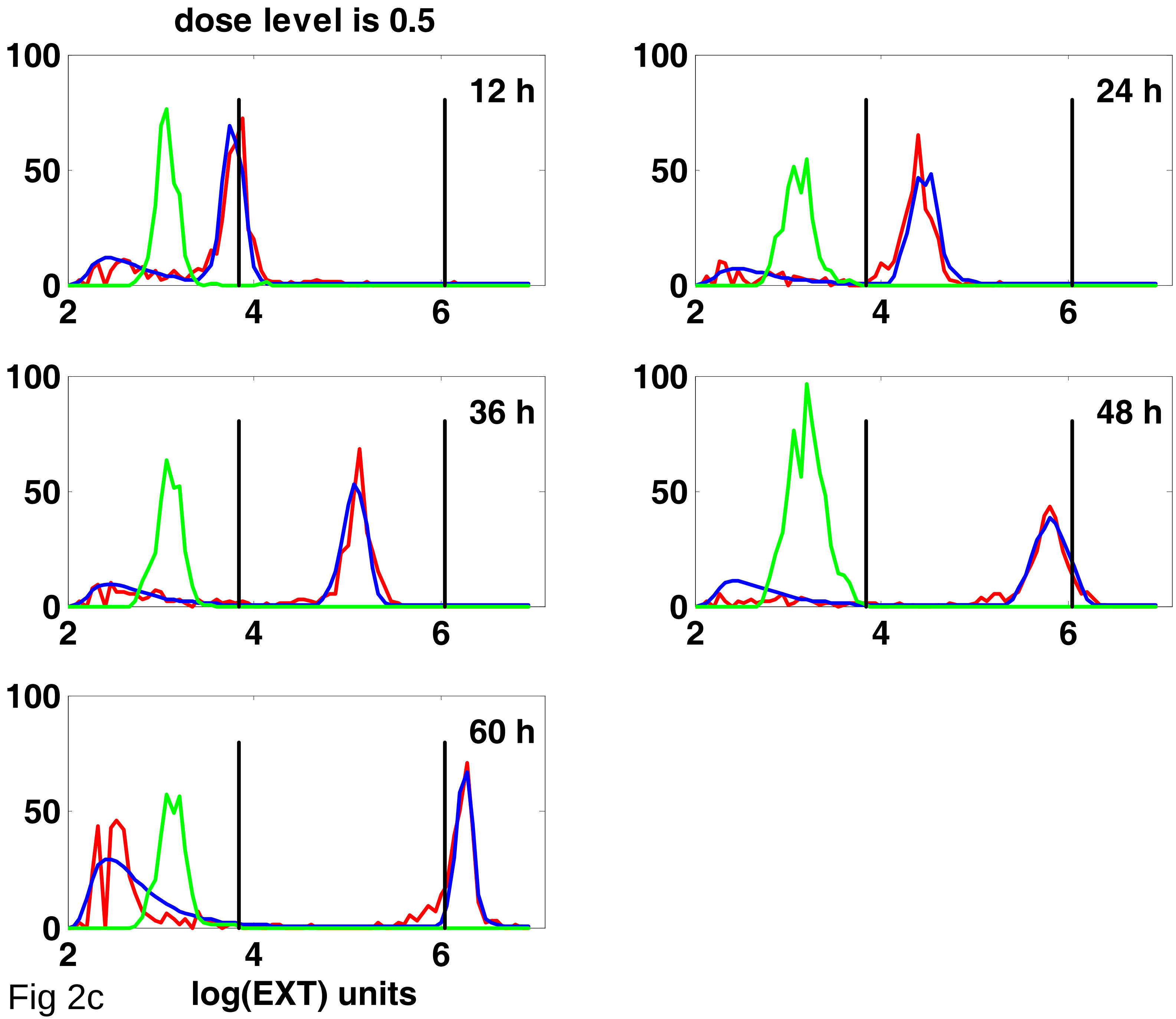

Suppl Fig 2c

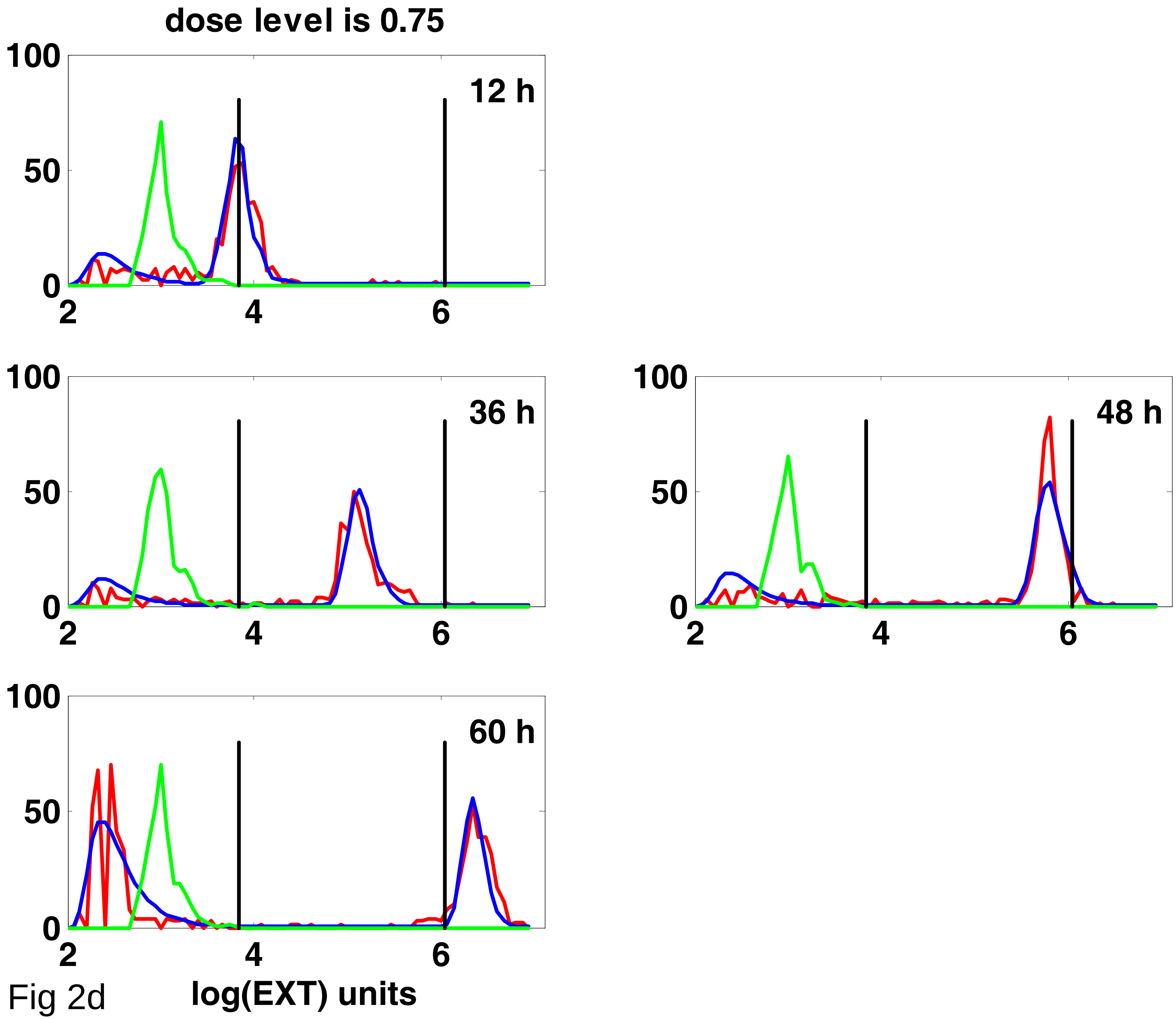

Suppl Fig 2d

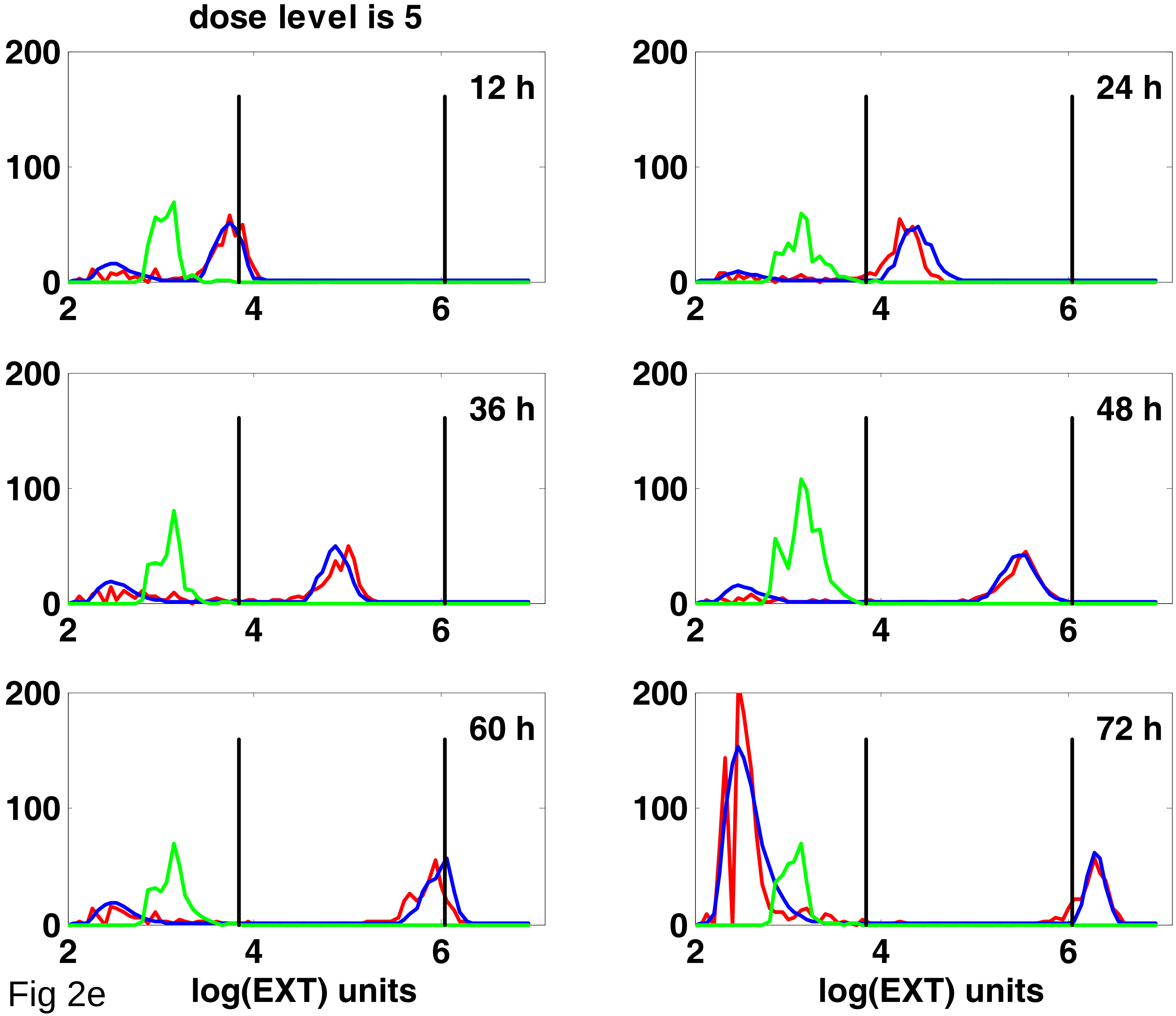
dose level is 5
200
100
0
2
4
6
12 h
24 h
36 h
48 h
60 h
72 h
log(EXT) units
log(EXT) units

Suppl Fig 2e

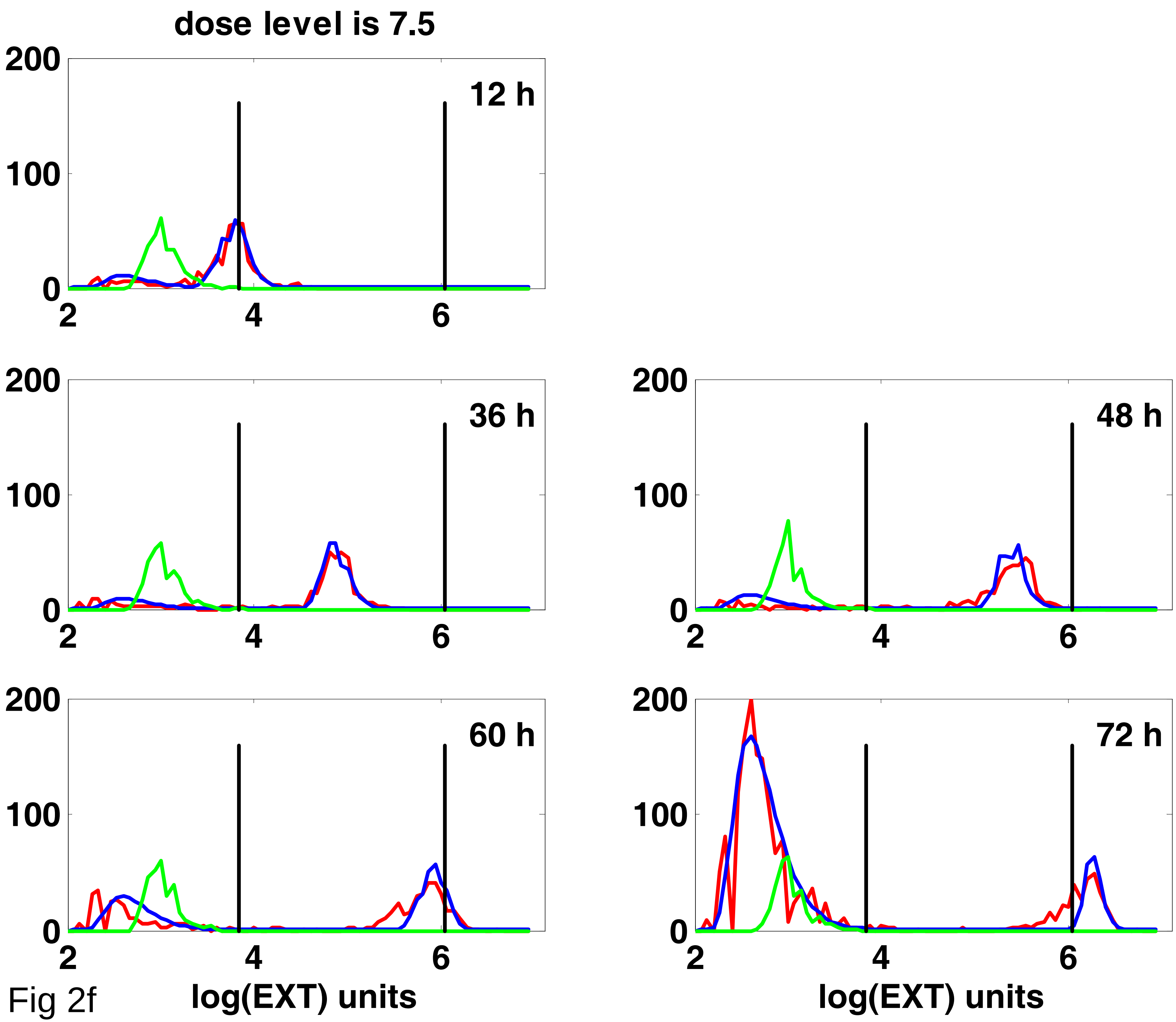
dose level is 7.5
200
100
0
2
4
6
12 h
36 h
48 h
60 h
72 h
log(EXT) units
log(EXT) units

Suppl Fig 2f

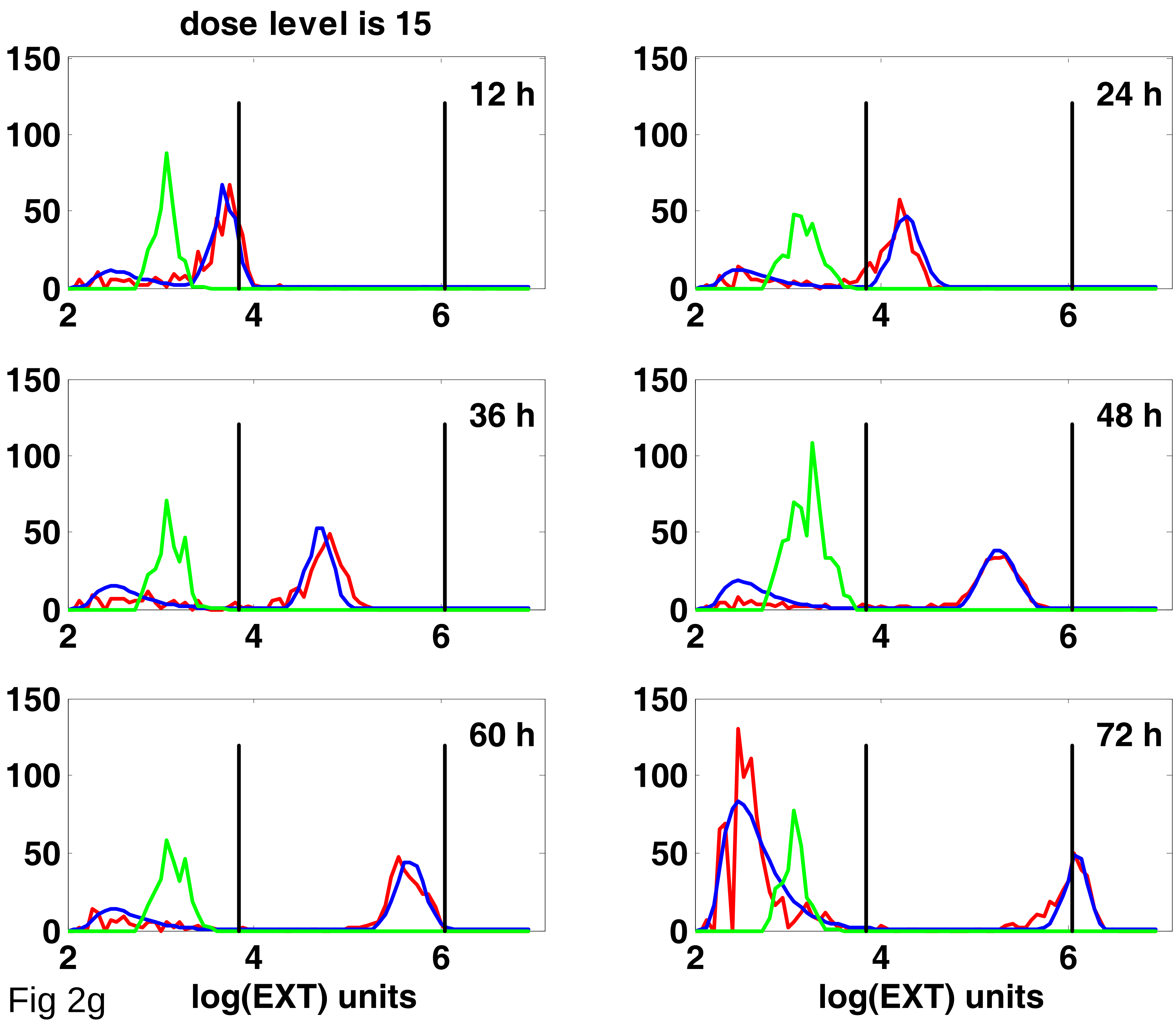
dose level is 15
12 h
24 h
36 h
48 h
60 h
72 h
150
100
50
0
2
4
6
log(EXT) units
log(EXT) units
Suppl Fig 2g

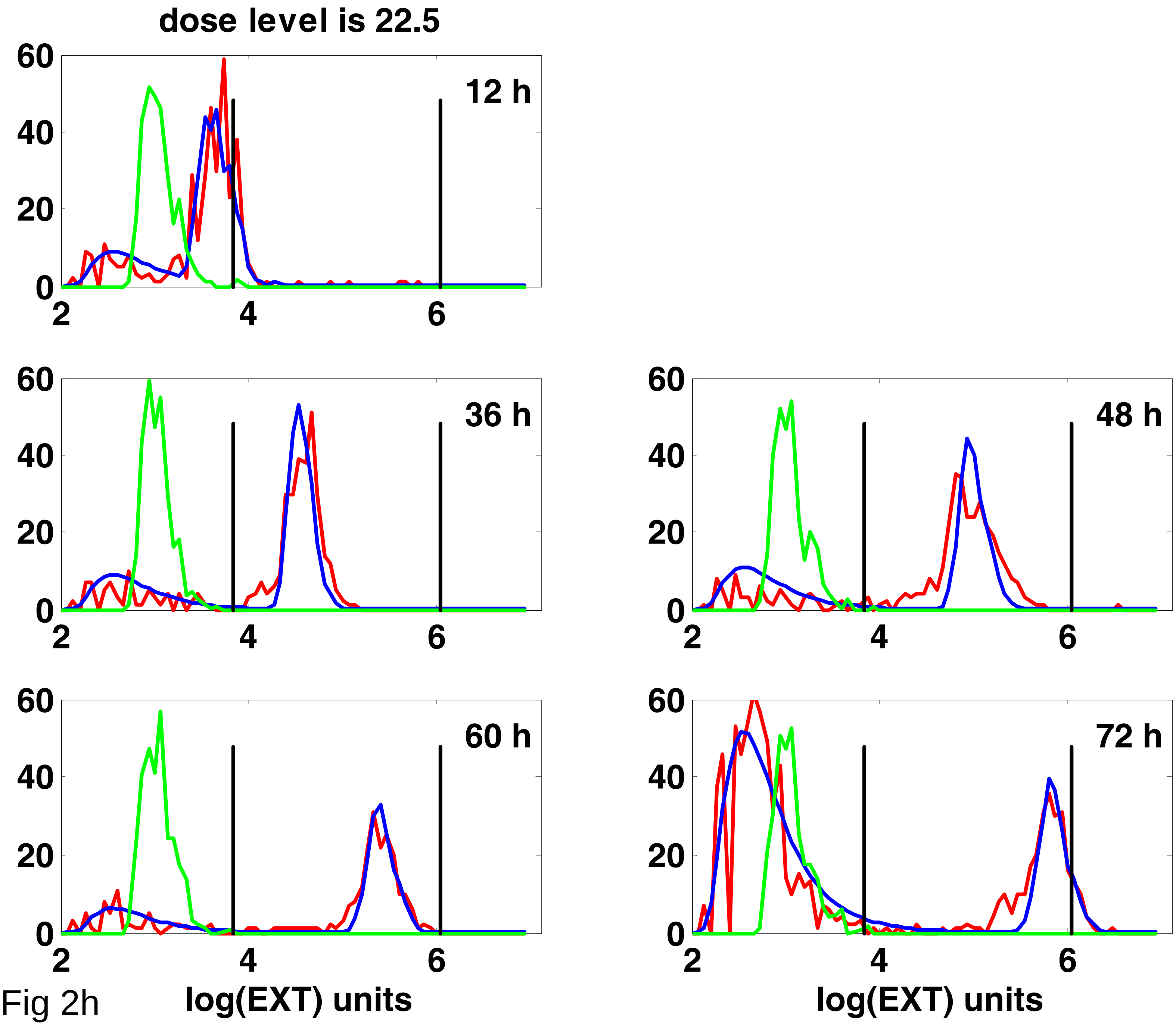
dose level is 22.5
12 h
36 h
48 h
60 h
72 h
60
40
20
0
2
4
6
log(EXT) units
log(EXT) units

Suppl Fig 2h

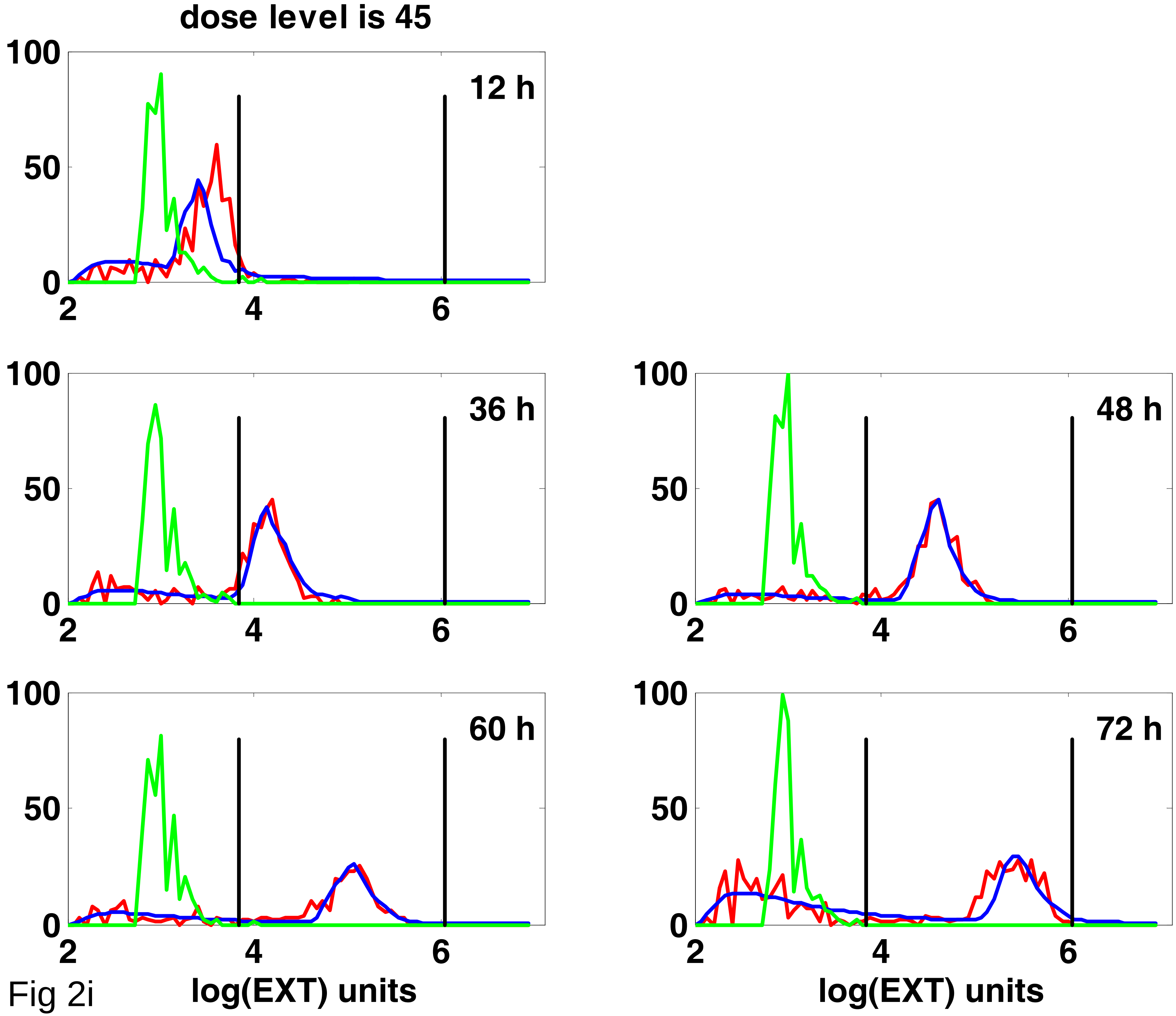
dose level is 45
12 h
36 h
48 h
60 h
72 h
100
50
0
2
4
6
log(EXT) units
log(EXT) units

Suppl Fig 2i

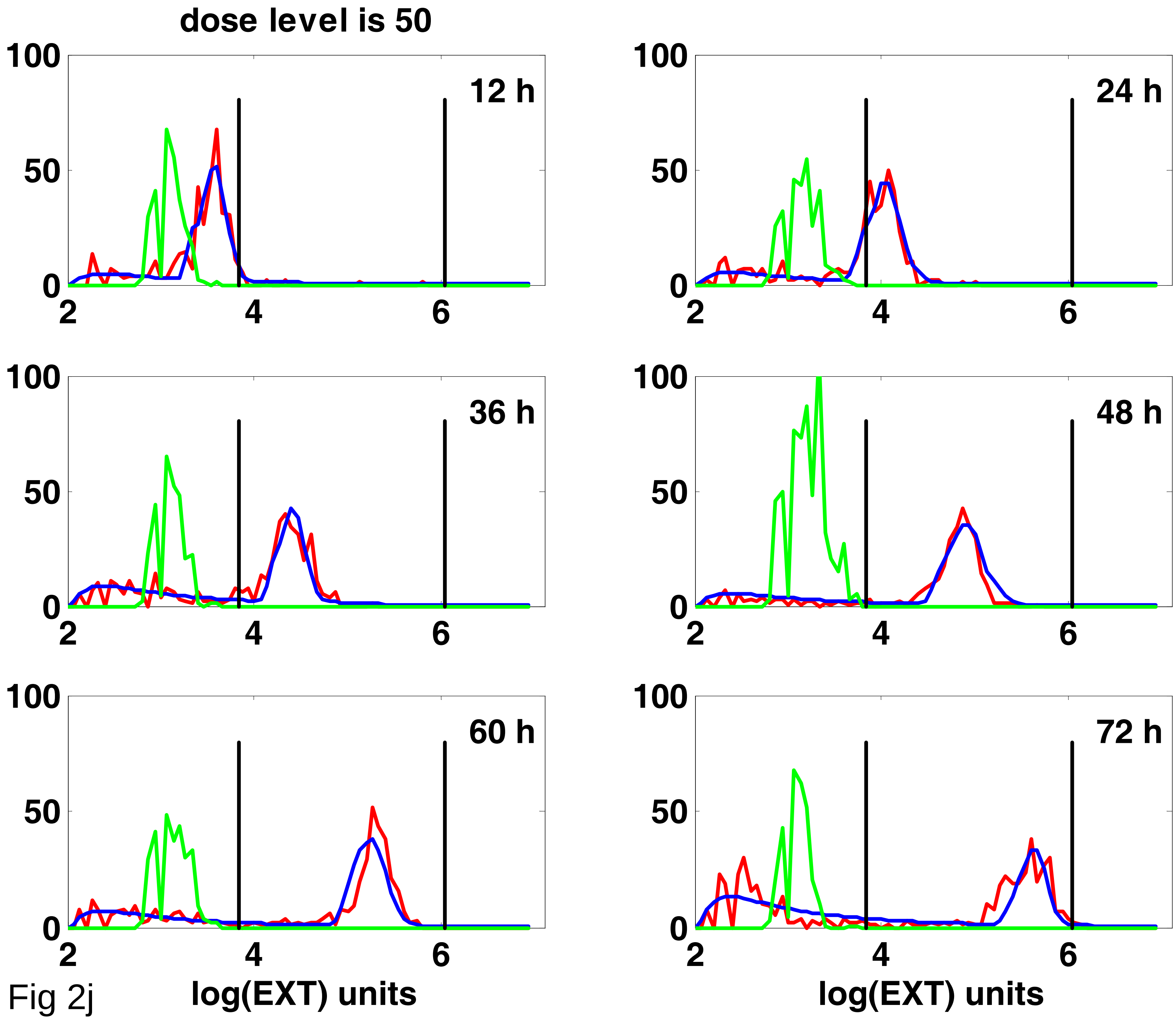
dose level is 50
100
50
0
2
4
6
12 h
24 h
36 h
48 h
60 h
72 h
log(EXT) units
log(EXT) units
Suppl Fig 2j
