# Supplementary material for: Application of a Mathematical Model to Describe the Effects of Chlorpyrifos on Caenorhabditis elegans Development
Source: PLoS One. 2009 Sep 15;4(9):e7024. doi: 10.1371/journal.pone.0007024 (PMC2737145; doi:10.1371/journal.pone.0007024)
Supplement: Supporting Information File S3 — Observed (red) and model-predicted (blue) distributions of log(TOF). The distribution of the loaded nematodes is shown in green. Extraneous noise was modeled as a lognormal distribution (black). Black vertical lines indicate change points, where growth rates changed. (0.08 MB PDF) [file pone.0007024.s003.pdf]

Supplementary File 3 - Observations and predictions of log(TOF) across all aspiration times and chlorpyrifos concentrations.

Observed frequencies (log(TOF)) of all aspirated measurements (red) and model estimated frequencies of maturing nematodes (blue). The distribution of the loaded nematodes is shown in green. Extraneous noise was modeled as a lognormal distribution (black). Black vertical lines indicate change points, where growth rates changed.

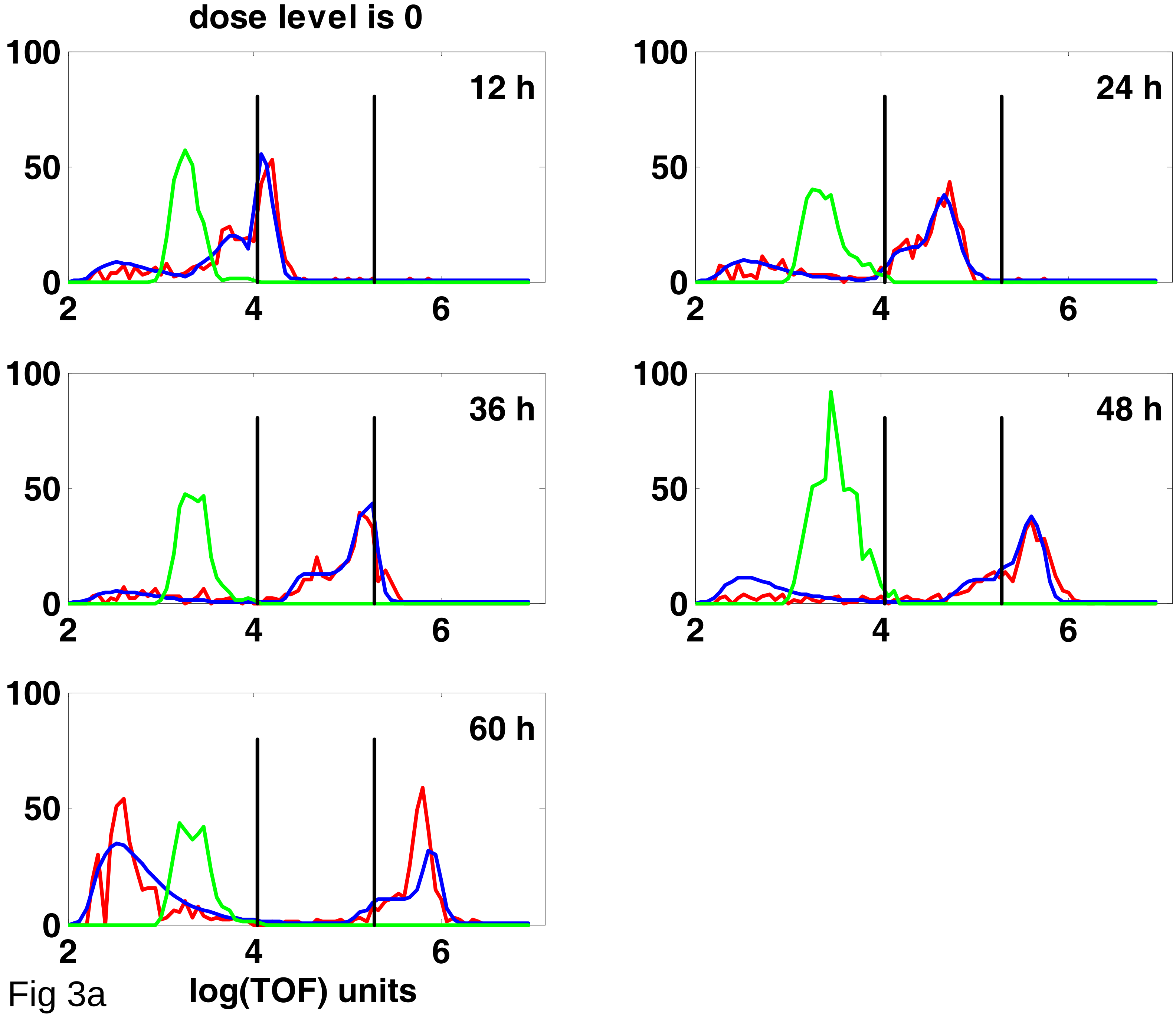

Suppl Fig 3a

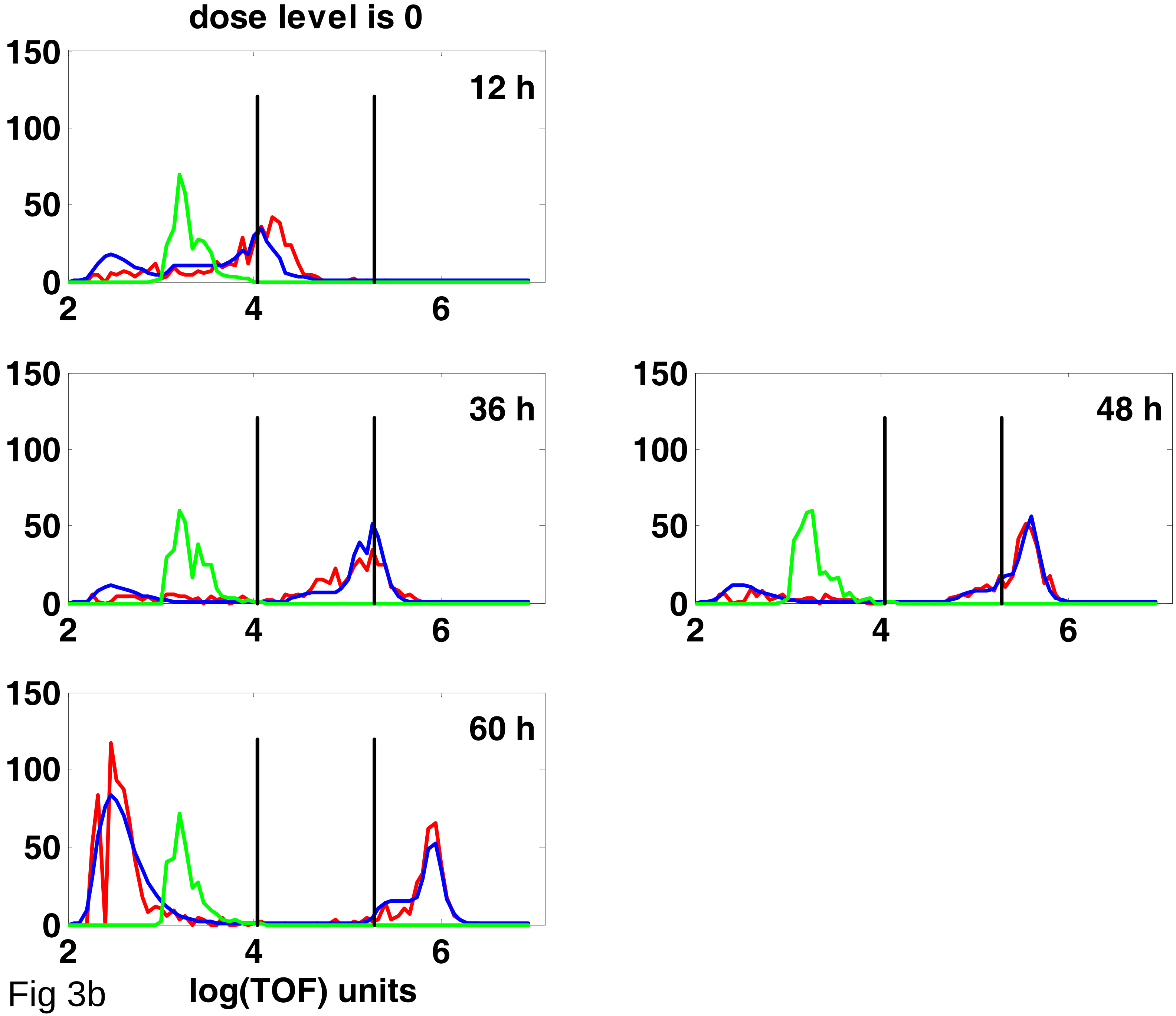

Suppl Fig 3b

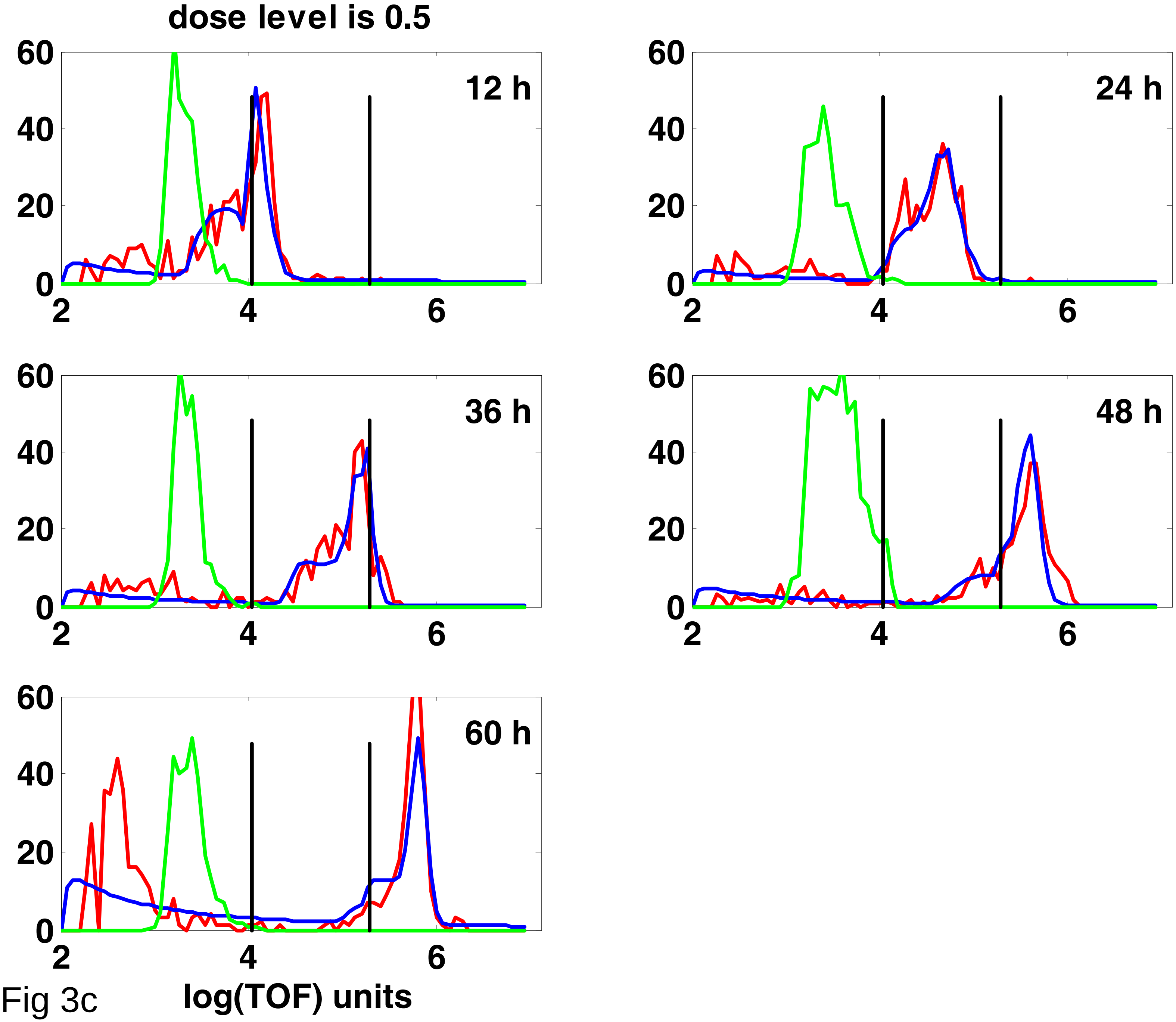

Suppl Fig 3c

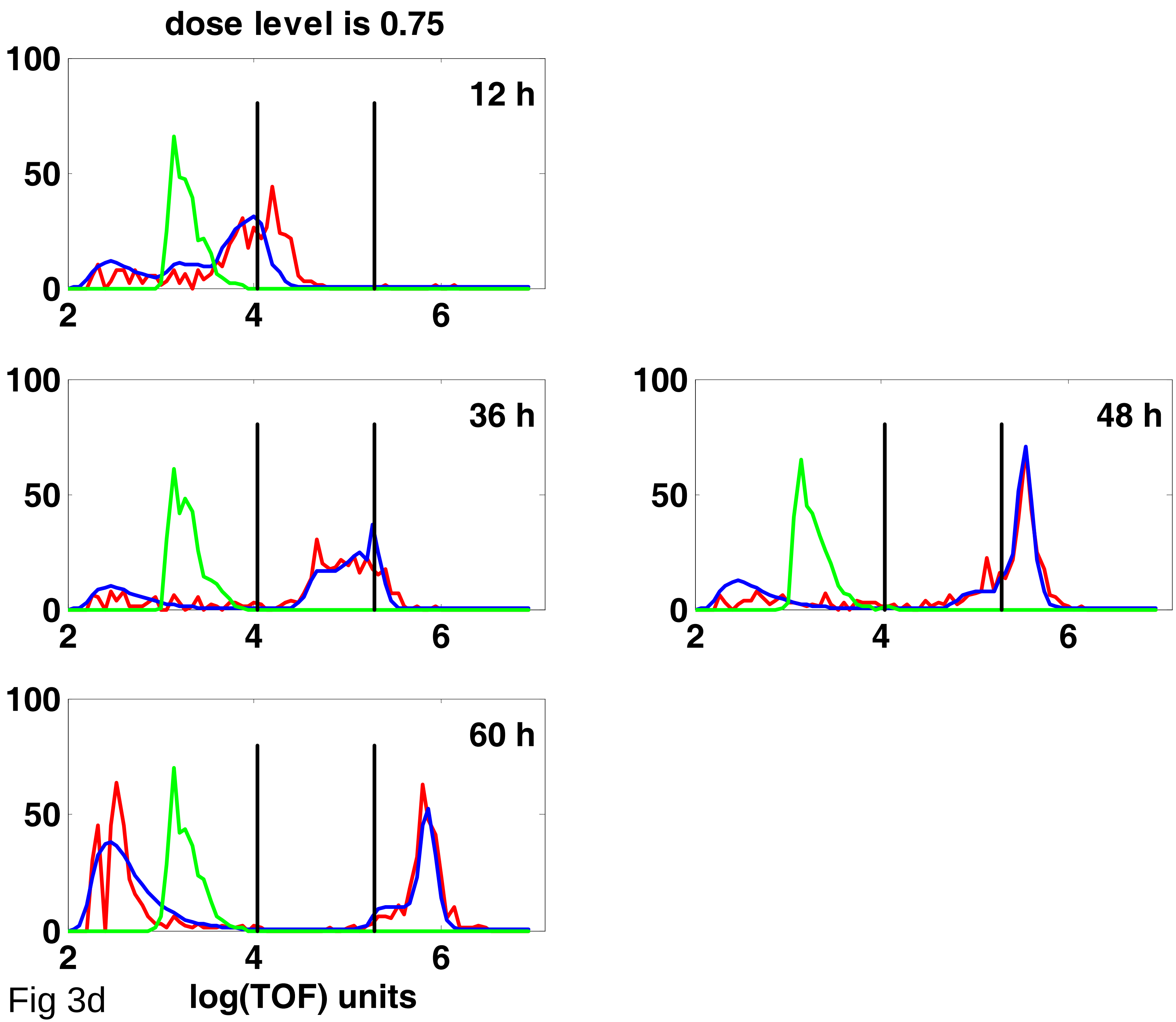
dose level is 0.75
100
50
0
2
4
6
12 h
100
50
0
2
4
6
36 h
100
50
0
2
4
6
48 h
100
50
0
2
4
6
60 h
log(TOF) units

Suppl Fig 3d

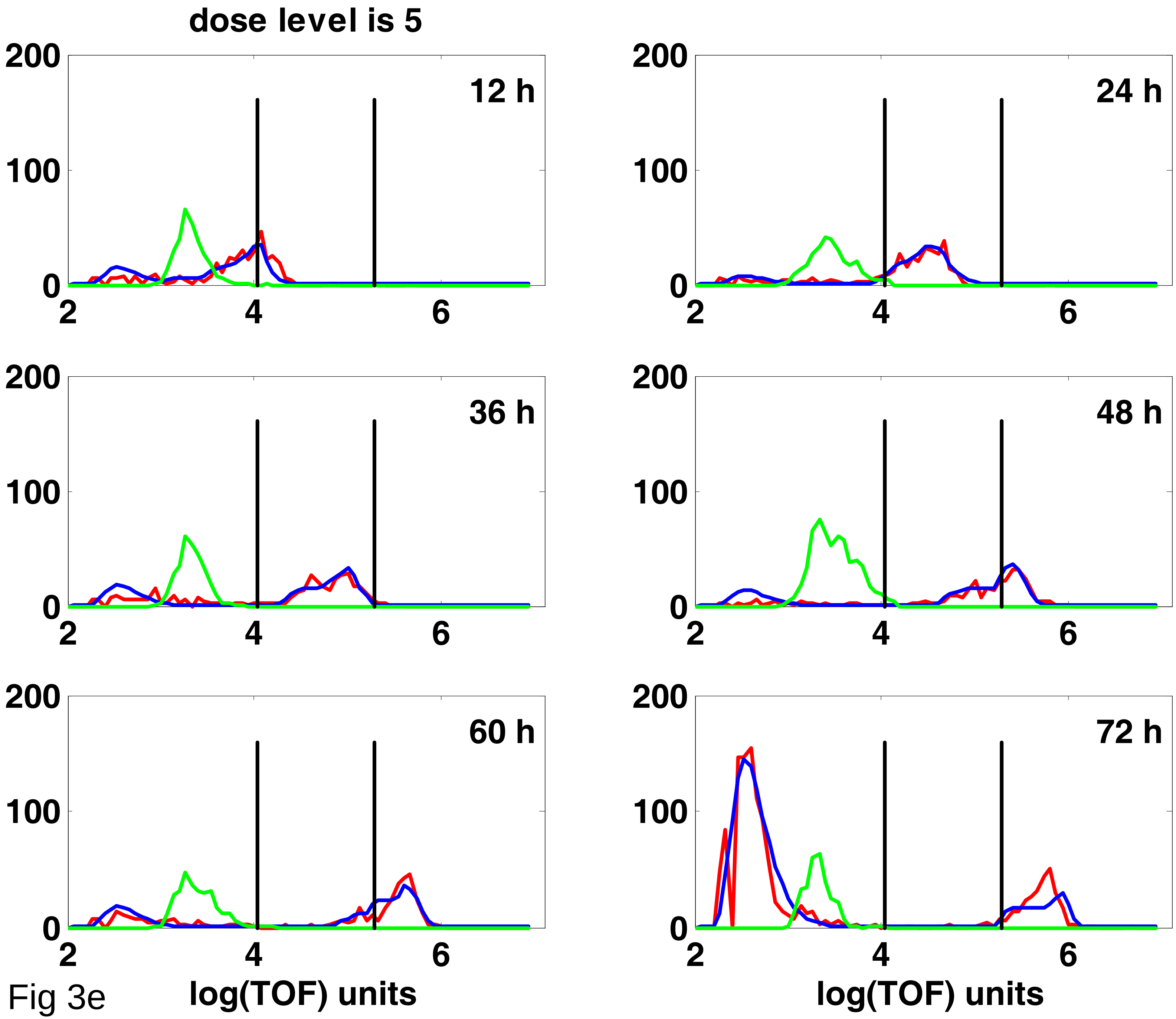
dose level is 5
12 h
24 h
36 h
48 h
60 h
72 h
200
100
0
2
4
6
log(TOF) units

Suppl Fig 3e

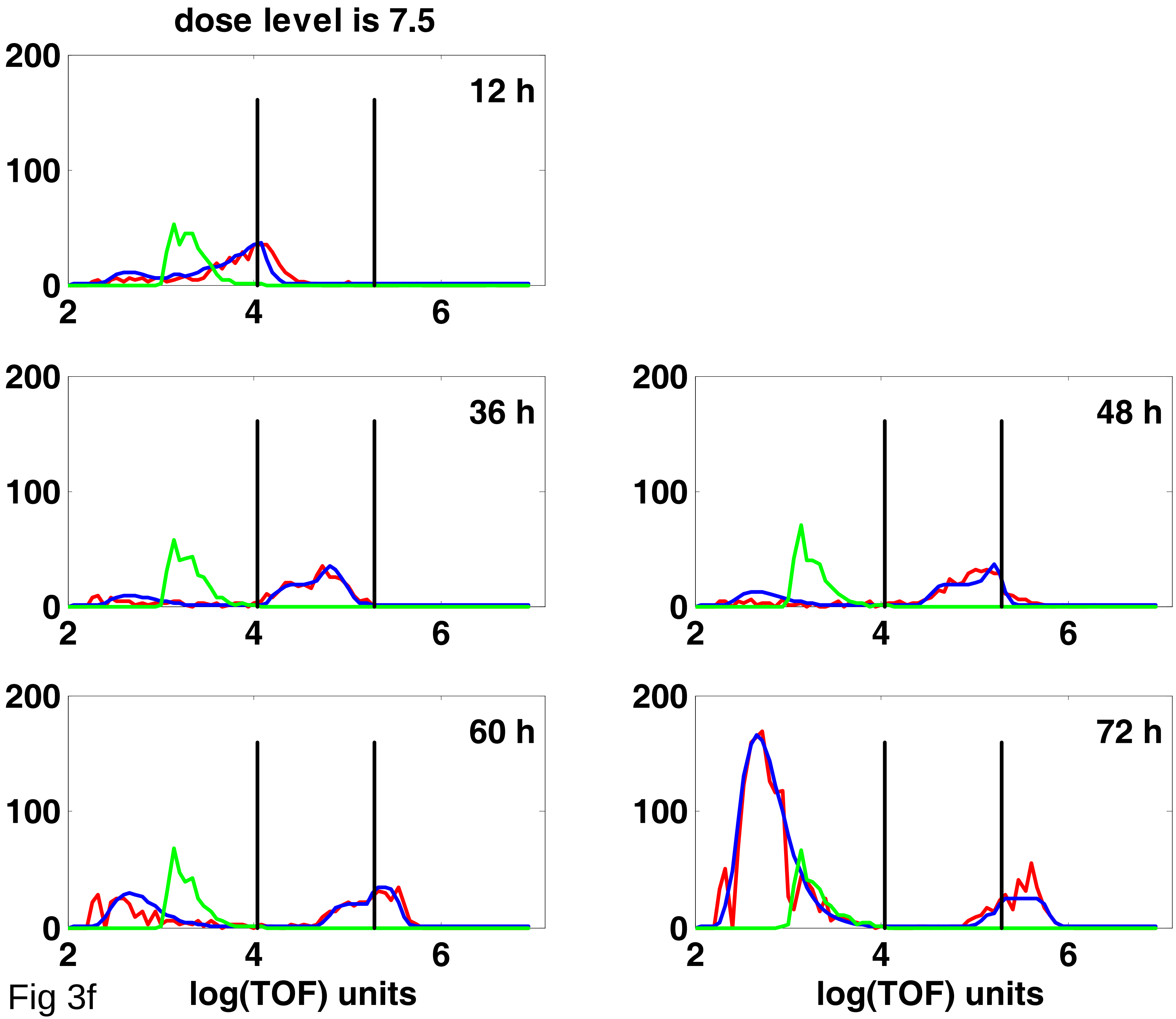

dose level is 7.5
200
100
0
2
4
6
12 h
36 h
48 h
60 h
72 h
log(TOF) units
log(TOF) units

Suppl Fig 3f

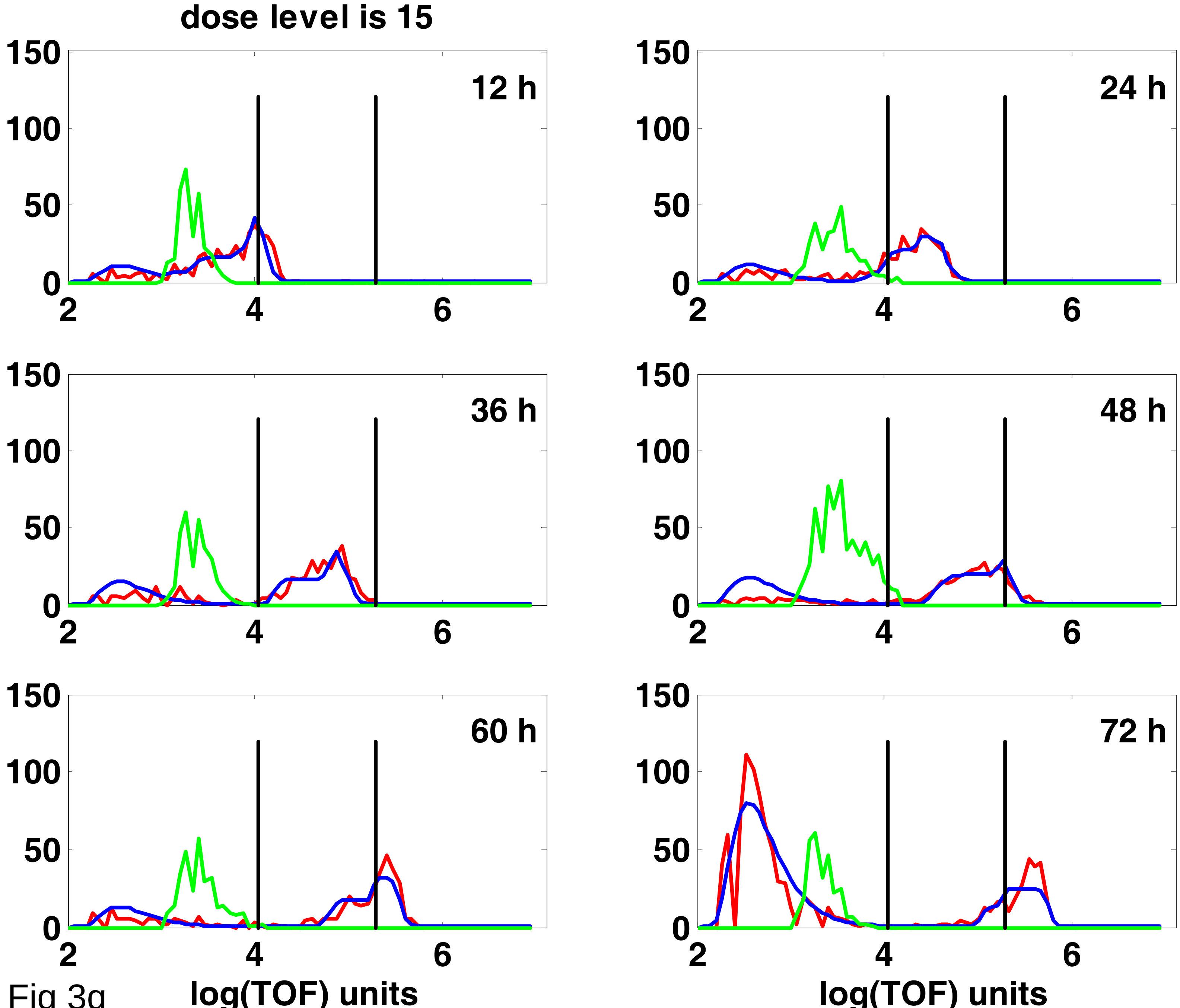
dose level is 15
12 h
24 h
36 h
48 h
60 h
72 h
150
100
50
0
2
4
6
log(TOF) units
log(TOF) units

Suppl Fig 3g

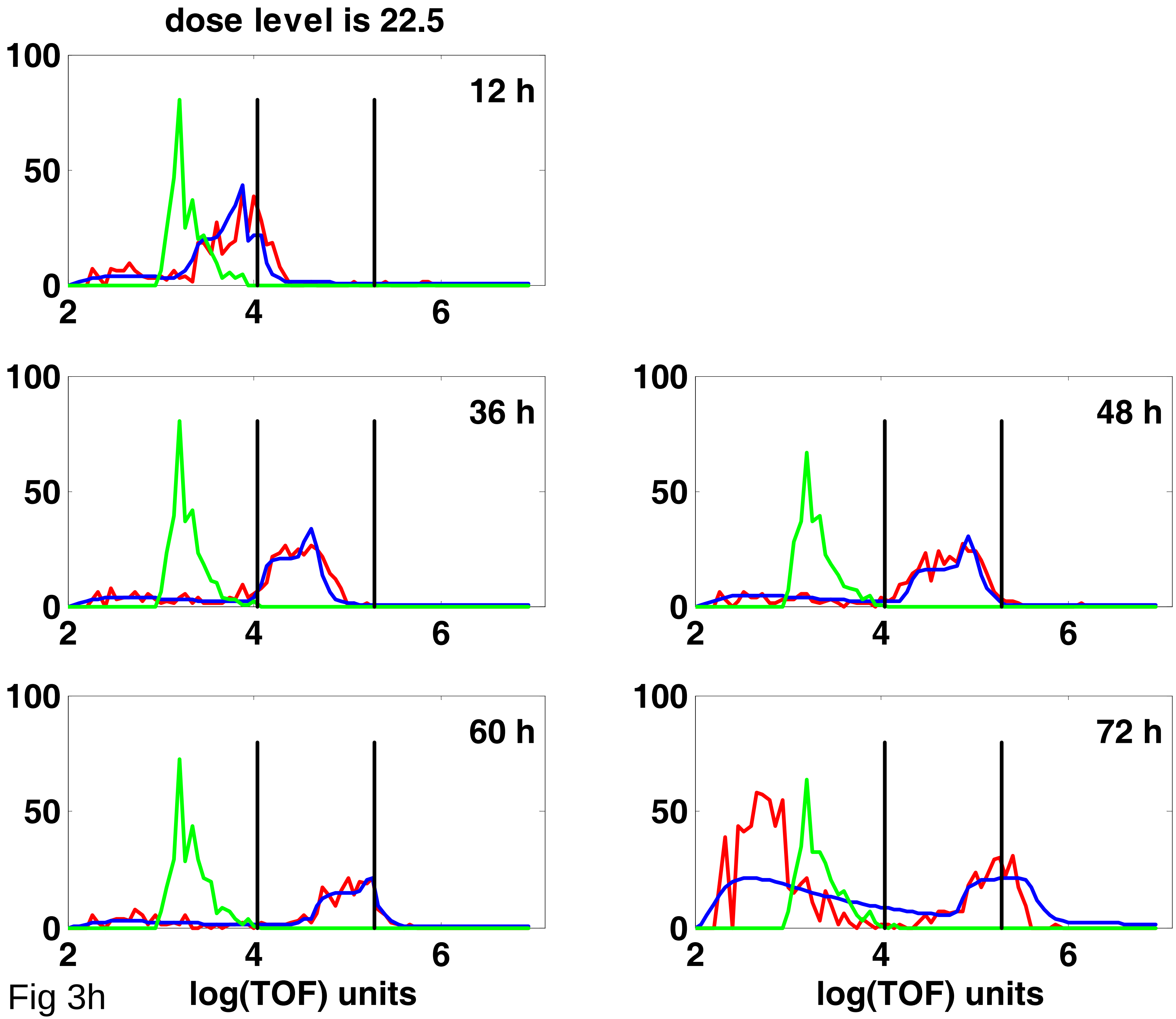
dose level is 22.5
12 h
36 h
48 h
60 h
72 h
100
50
0
2
4
6
log(TOF) units
log(TOF) units

Suppl Fig 3h

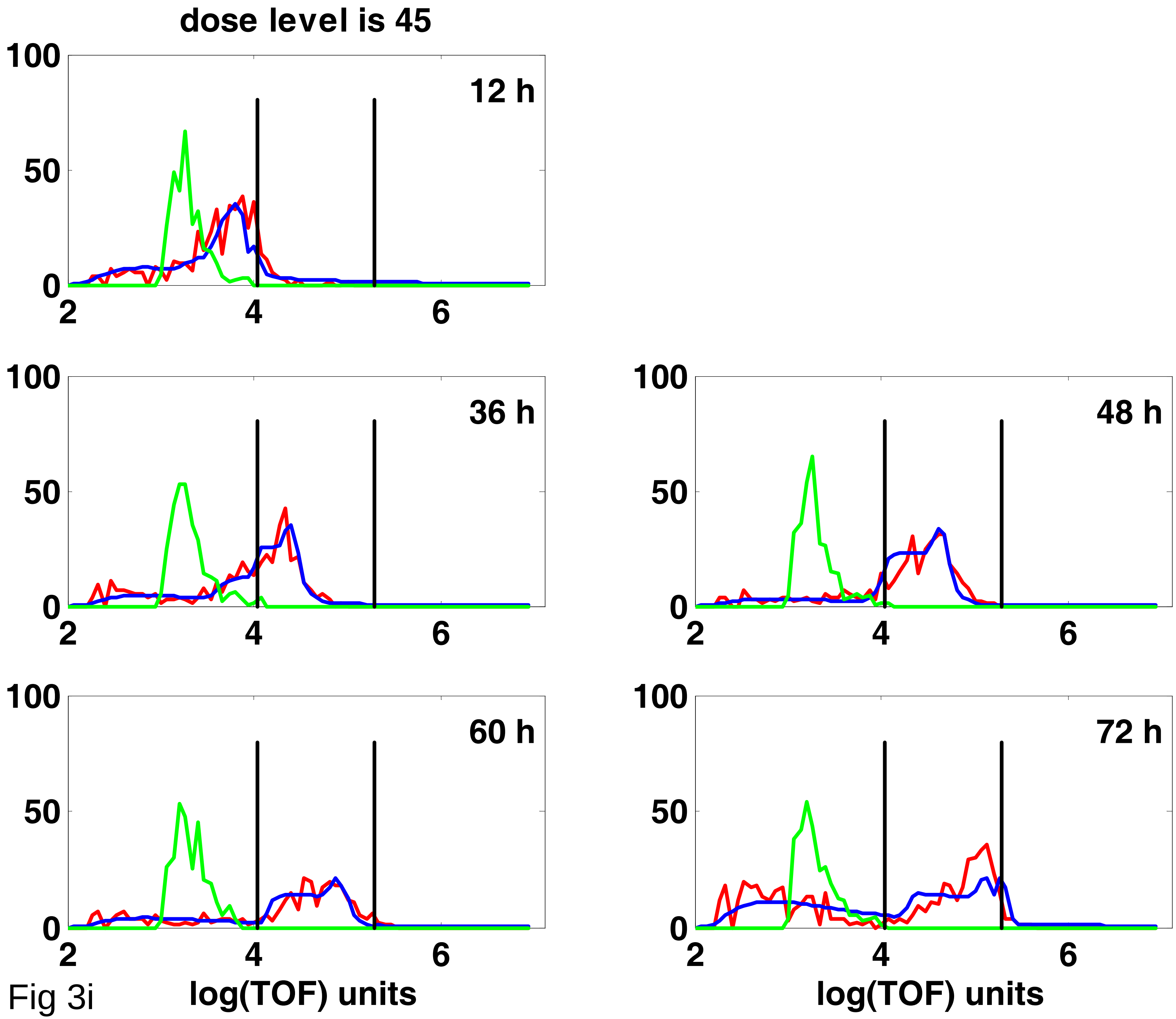
dose level is 45
100
50
0
2
4
6
12 h
36 h
48 h
60 h
72 h
log(TOF) units
log(TOF) units

Suppl Fig 3i
