# Supplementary material for: Application of a Mathematical Model to Describe the Effects of Chlorpyrifos on Caenorhabditis elegans Development
Source: PLoS One. 2009 Sep 15;4(9):e7024. doi: 10.1371/journal.pone.0007024 (PMC2737145; doi:10.1371/journal.pone.0007024)
Supplement: Supporting Information File S4 — Tables (0.07 MB PDF) [file pone.0007024.s004.pdf]

SUPPLEMENTARY FILE 4 - TABLES

Table A1. Noise parameters for log(EXT) fit

Concentration (μM)	Lognormal Parameters	
0	-0.4967	0.5553
0	-0.7683	0.4176
0.50	-0.4685	0.5910
0.75	-0.7345	0.5130
5.00	-0.6329	0.3554
7.50	-0.3851	0.3648
15.0	-0.5255	0.4713
22.5	-0.3196	0.5186
30.0	-0.1526	0.9995
45.0	0.0647	0.8632
50.0	0.0205	1.0000

Table A2. Estimated numbers of aspirated nematodes from fits to log(EXT) data

Concentration (μM)	Time (hours)					
	**12**	**24**	**36**	**48**	**60**	**72**
0	300	277	298	270	216	*
0	300	*	300	300	293	*
0.50	286	268	259	260	232	*
0.75	294	*	261	300	236	*
5.00	259	291	257	279	269	226
7.50	300	*	292	275	286	229
15.0	283	264	257	264	260	222
22.5	269	*	243	227	170	209
30.0	242	274	198	270	256	196
45.0	190	*	256	278	187	210
50.0	261	266	208	251	242	175

*The model was not fit to these dose/time combinations

Table A3. Estimated transfer probabilities for log(EXT) fit across all times

Concentration (μM)	Probability		
	10 bins	11 bins	12 bins
0	0.2862	0.5849	0.1289
0	0.3590	0.3726	0.2684
0.50	0.2036	0.6863	0.1100
0.75	0.3092	0.4312	0.2595
5.00	0.5881	0.0151	0.3968
7.50	0.4768	0	0.5232
15.0	0.4905	0.0535	0.4559
22.5	0.5077	0	0.4923
30.0	0.5213	0.0615	0.4172
45.0	0.2255	0.1705	0.6040
50.0	0.3906	0.1950	0.4143

Table A4. Number of bins estimated for each section delineated by change points across all times

Concentration (μM)	Section (log(EXT))		
	2.000 - 3.828	3.828 - 6.039	6.039 – 7.000
0	26	38	27
0	24	36	18
0.50	28	38	26
0.75	24	37	22
5.00	29	44	32
7.50	26	46	37
15.0	32	51	28
22.5	32	57	31
30.0	39	54	34
45.0	57	58	16
50.0	44	60	34

Table A5. Noise parameters for log(TOF) fit

Concentration (μM)	Lognormal Parameters	
0	-0.3441	0.5360
0	-0.5845	0.4040
0.50	0.4013	1.5000
0.75	-0.5463	0.5087
5.00	-0.4998	0.3250
7.50	-0.2906	0.3305
15.0	-0.4111	0.4250
22.5	0.2864	0.8927
30.0	0.1791	0.8101
45.0	0.3338	0.7187

Table A6. Estimated transfer probabilities for log(TOF) fit across all times

Concentration (μM)	Probability		
	10 bins	11 bins	12 bins
0	0.3625	0.0000	0.6375
0	0.3694	0.0725	0.5582
0.50	0.5008	0.0000	0.4992
0.75	0.2886	0.1129	0.5985
5.00	0.4092	0.0000	0.5908
7.50	0.3850	0.0730	0.5420
15.0	0.0234	0.2660	0.7106
22.5	0.0968	0.0059	0.8974
30.0	0.2075	0.0000	0.7925
45.0	0.4903	0.0390	0.4708

Table A7. Estimated number of bins in each section as delineated by change points for log(TOF) measurements across all times

Concentration (μM)	Section (log(TOF))		
	2.000 - 4.035	4.035 - 5.280	5.280 – 7.000
0	25	27	57
0	32	21	59
0.50	27	25	68
0.75	30	24	62
5.00	31	29	70
7.50	30	33	81
15.0	32	35	87
22.5	37	44	64
30.0	38	37	73
45.0	41	56	163

Table A8. Fraction of aspirated nematodes with some degree of curling while being aspirated*

Concentration (μM)	Section (log(TOF))		
	2.000 - 4.035	2.000 - 4.035	2.000 - 4.035
0	0.9500	0.4794	0.5584
0	0.6713	0.5083	0.4790
0.50	0.9500	0.4579	0.4551
0.75	0.7487	0.6530	0.3838
5.00	0.6081	0.5703	0.7202
7.50	0.5508	0.6354	0.9228
15.0	0.5834	0.6621	0.9865
22.5	0.5308	0.6522	0.9360
30.0	0.6087	0.6877	0.9493
45.0	0.2173	0.7620	0.0142

*Fraction of all nematodes with some degree of curling while being loaded is 0.32.

Table A9. Minimum fraction of length due to curling of log(TOF) measurement of aspirated nematodes across all times *

Concentration (μM)	Section (log(TOF))		
	2.000 - 4.035	2.000 - 4.035	2.000 - 4.035
0	0.8775	0.6735	0.6691
0	0.6034	0.9902	0.7171
0.50	0.7297	0.7255	0.6689
0.75	0.5879	0.7981	0.7177
5.00	0.6154	0.7367	0.7027
7.50	0.6092	0.6825	0.6854
15.0	0.5925	0.6663	0.6688
22.5	0.7286	0.6716	0.7179
30.0	0.6974	0.7505	0.6330
45.0	0.7509	0.5842	0.9525

*Minimum fraction of length for all loaded nematodes is 0.932
