# Supplementary material for: Application of a Mathematical Model to Describe the Effects of Chlorpyrifos on Caenorhabditis elegans Development
Source: PLoS One. 2009 Sep 15;4(9):e7024. doi: 10.1371/journal.pone.0007024 (PMC2737145; doi:10.1371/journal.pone.0007024)
Supplement: Supporting Information File S5 — Negative exponential functions fit to growth rates. (0.01 MB PDF) [file pone.0007024.s005.pdf]

SUPPLEMENTARY FILE 5

EQUATIONS CORRESPONDING TO FIGURES 5 AND 6

**Log(EXT) Growth Rates**	**Log(TOF) Growth Rates**
$g_1 = 0.0366e^{-0.0344 dose} + 0.0303$	$g_1 = 0.045e^{-0.0142 dose} + 0.0222$
$g_2 = 0.0234e^{-0.0542 dose} + 0.0303$	$g_2 = 0.026e^{-0.0596 dose} + 0.0222$
$g_3 = 0.0091e^{-0.5522 dose} + 0.0303$	$g_3 = 0.0054e^{-1.2086 dose} + 0.0222$
Expected time to 1st change point = 9.8 + 0.21 dose	Expected time to 1st change point = 9.8 + 0.09 dose
Expected time to 2nd change point = 53.5 + 0.73 dose	Expected time to 2nd change point = 36.5 + 0.73 dose
